# YAP establishes epiblast responsiveness to inductive signals for germ cell fate

**DOI:** 10.1242/dev.199732

**Published:** 2021-10-19

**Authors:** Saya Kagiwada, Shinya Aramaki, Guangming Wu, Borami Shin, Eva Kutejova, David Obridge, Kenjiro Adachi, Jeffrey L. Wrana, Karin Hübner, Hans R. Schöler

**Affiliations:** 1 Department of Cell and Developmental Biology, Max Planck Institute for Molecular Biomedicine, Münster 48149, Germany; 2 Guangzhou Regenerative Medicine and Health Guangdong Laboratory, Luoxuan Avenue, Haizhu District, 510320 Guangzhou, PRC; 3 Department of Cancer Biology, Centre for Systems Biology, Lunenfeld Tanenbaum Research Institute, Mount Sinai Hospital, Toronto, ON M5G 1X5, Canada; 4 Department of Molecular Genetics, University of Toronto, Toronto, ON M5S 1A8, Canada; 5 Medical Faculty, University of Münster, Münster 48149, Germany

**Keywords:** EpiSCs, PGCs, Hippo pathway, Pluripotent stem cells, Mouse

## Abstract

The germ cell lineage in mammals is induced by the stimulation of pluripotent epiblast cells by signaling molecules. Previous studies have suggested that the germ cell differentiation competence or responsiveness of epiblast cells to signaling molecules is established and maintained in epiblast cells of a specific differentiation state. However, the molecular mechanism underlying this process has not been well defined. Here, using the differentiation model of mouse epiblast stem cells (EpiSCs), we have shown that two defined EpiSC lines have robust germ cell differentiation competence. However, another defined EpiSC line has no competence. By evaluating the molecular basis of EpiSCs with distinct germ cell differentiation competence, we identified YAP, an intracellular mediator of the Hippo signaling pathway, as crucial for the establishment of germ cell induction. Strikingly, deletion of YAP severely affected responsiveness to inductive stimuli, leading to a defect in WNT target activation and germ cell differentiation. In conclusion, we propose that the Hippo/YAP signaling pathway creates a potential for germ cell fate induction via mesodermal WNT signaling in pluripotent epiblast cells.

## Abstract (precis)

**Summary:** YAP, an intracellular mediator of the Hippo signaling pathway, establishes epiblast competency for germ cell differentiation through activation of the WNT pathway.

## INTRODUCTION

Germ cells are highly specialized cells that enable the transmission of genetic information and the development of a new complete organism, linking the successive generations of organisms. Supposedly, in most mammalian species, including mice and humans, germ cells are zygotically induced from the pluripotent epiblast.

Mouse germ cell specification starts in the epiblast at the gastrulation stage, which is induced by a combination of signaling molecules (Ginsburg et al., 1990; Ohinata et al., 2005). In mice, BMP/SMAD signaling has been extensively studied and it has been demonstrated that the pathway is essential for the induction of germ cells and other neighboring somatic lineages in nascent mesoderm derived from pluripotent epiblast (Lawson et al., 1999; Tremblay et al., 2001; Winnier et al., 1995). More recently, the WNT/β-catenin signaling pathway has been shown to regulate germ cell fate in a cooperative manner (Aramaki et al., 2013; Ohinata et al., 2009), in addition to its well-defined function in pan-mesoderm development (Huelsken et al., 2000; Liu et al., 1999). Therefore, these previous studies suggest that germ cell fate is induced together with other somatic mesoderm lineages downstream of common mesodermal signaling at the onset of gastrulation. Indeed, recent studies have shown that mesodermal factors, such as T (brachyury), are required for germ cell specification and directly regulate germ cell determinants in the specific pluripotent cell state, suggesting that there is context-dependent induction of mesodermal lineages, including the germline and somatic cell lineages (Aramaki et al., 2013, 2021; Chen et al., 2017; Kojima et al., 2017).

The signaling pathways described above have been consistently shown to promote *in vitro/ex vivo* germ cell induction from pluripotent cell models. Recombinant BMP4, and its combination with other signaling molecules, has been shown to induce germ cell fate from several embryonic cell models with responsiveness to signals or competence for germ cell differentiation. For example, germ cell specification-related genes are noticeably induced by BMP4 stimulation of epiblast stem cells (EpiSCs) derived from post-implantation mouse epiblast (Tesar et al., 2007). Subsequent studies have shown that pre-gastrulating mouse epiblast cells at around embryonic day (E) 6.0 and a specific state of differentiating mouse embryonic stem cells (ESCs) known as epiblast-like cells (EpiLCs) have a marked responsiveness to cytokines for germ cell induction (Hayashi et al., 2011; Ohinata et al., 2009). Although these studies have suggested that a distinct pluripotent cell state exhibits responsiveness to the signaling molecules required for germ cell fate induction, the way this specific state is established and the mechanism underlying this process are not well defined.

The Hippo signaling pathway is a potent regulator of cell differentiation in various developmental contexts (Meng et al., 2016), although it is not known whether it has any role in germ cell fate induction. The Hippo downstream factors Yes-associated protein 1 (YAP; also known as YAP1 and YAP65) and WW domain-containing transcription regulator 1 (WWTR1; also known as TAZ), function as transcriptional co-activators by binding to TEA domain (TEAD) proteins, leading to the formation of an active transcriptional complex that regulates gene expression. Downstream of Hippo signaling, YAP and TAZ are inactivated by phosphorylation and subsequent exclusion from the nucleus, which results in proteolytic degradation.

In our previous study, distinct EpiSC lines were derived and characterized, displaying characteristics typical of EpiSCs, such as teratoma formation and expression of core pluripotency factors (Bernemann et al., 2011). Interestingly, however, they also exhibited different pluripotency features: specific gene expression profile, reprogramming efficiency, and differentiation ability. Here, by taking advantage of such characteristic differences between EpiSC lines, we explored the regulatory mechanism underlying epiblastic responsiveness to signals required for germ cell fate induction.

## RESULTS

### E3 and T9 EpiSC lines have germ cell competence

Our previous studies established several EpiSC lines (Bernemann et al., 2011; Greber et al., 2010). Of these, E3 male EpiSCs (E3) and E5 male EpiSCs (E5) represent relatively early post-implantation epiblast cells. However, the ability of these EpiSC lines to differentiate into germ cells (germ cell competence) was not determined. By contrast, T9 female EpiSCs (T9), also characterized as early post-implantation epiblast, show induction of germ cell genes in response to BMP4 (Tesar et al., 2007). Moreover, previous studies showed a time window from E5.5 to E6.5 for germ cell competence in epiblast cells *in vivo* (Ohinata et al., 2009) and in EpiLCs, a specific differentiated state derived from naïve mouse ESCs (2iL ESCs) (Hayashi et al., 2011).

**Fig. 1.**
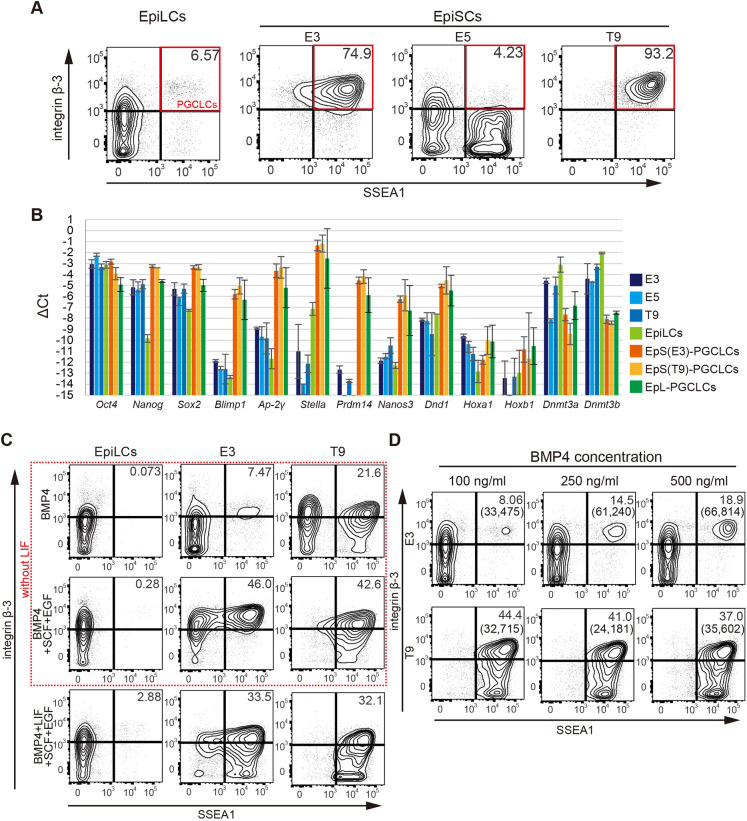
**E3 and T9 EpiSC lines have germ cell competence.** (A) Representative FACS plots of SSEA1 and integrin β-3 expression in cultured aggregates derived from EpiLCs and the E3, E5 and T9 cell lines stimulated with cytokines (BMP4, SCF, EGF and LIF) on day 6 (three independent experiments). The percentage of induced PGCLC population is shown on each plot. Red square indicates the sorting gate of PGCLCs for Q-PCR in B. See also Fig. S1B. (B) Expression of the pluripotent genes and the germ cell-related genes in EpiLCs, EpiSCs and sorted PGCLCs. Gene expression of the indicated genes was measured by Q-PCR. For each gene examined, the ΔCT from the average CT values of the two independent housekeeping genes, *Arbp* and *Ppia*, was calculated. The average value from two independent experiments (log_2_ scale) is shown ±s.d. (C) Representative FACS plots of SSEA1 and integrin β-3 expression in cultured aggregates derived from EpiLCs, and the E3 and T9 cell lines under the conditions indicated on day 6 (two independent experiments). The percentage of induced PGCLC population is shown on each plot. The red dashed square indicates the conditions without LIF. See also Fig. S1F. (D) Representative FACS plots of SSEA1 and integrin β-3 expression in cultured aggregates derived from the E3 and T9 cell lines under conditions without LIF and with the indicated concentration of BMP4 on day 6 (two independent experiments). The concentration of BMP4 is shown at the top. The starting number of EpiSCs was 2000 cells/aggregate, and 30 cell aggregates were collected for FACS analysis. The percentage and the number (parentheses) of the induced PGCLC population are shown on each plot. See also Fig. S1G-I.

Based on these previous studies, we examined the germ cell competence of E3, E5 and T9 lines by culturing them under primordial germ cell-like cell (PGCLC) induction conditions (Fig. S1A). We observed induction of a population of PGCLCs that were positive for both integrin β-3 and SSEA1 (FUT4) in the aggregates derived from the E3 and T9 but not E5 lines (Fig. 1A, Fig. S1B). In these two different EpiSC lines, PGCLCs were induced with robust efficiency, which was higher than that for EpiLCs (Fig. S1B), suggesting that the E3 and T9 lines retain competence for PGCLC induction even through several passages. We performed Q-PCR analysis in the sorted fraction of PGCLCs (Fig. 1A), which revealed that germ cell-related genes were induced in the PGCLCs derived from EpiSCs (EpS-PGCLCs) at levels comparable to those in PGCLCs induced from EpiLCs (EpL-PGCLCs) (Fig. 1B).

A previous study has shown that EpiSCs cultured under a specific condition [N2B27 medium with 20% knockout serum replacement (KSR), 20 ng/ml activin A and 12 ng/ml basic fibroblast growth factor (bFGF)] are not able to differentiate into germ cells (Hayashi et al., 2011). Under the same culture condition, E3 and T9 showed a lower level of germ cell induction in response to cytokines (Fig. S1C,D), suggesting that undefined factors in conditioned medium (CM) maintain competence. Another recent study has demonstrated the derivation of formative stem cells (FSCs) from mouse epiblast (Kinoshita et al., 2021). Unlike primed EpiSCs cultured under the condition of the original study (Hayashi et al., 2011), FSCs noticeably maintained germ cell competence for induction of PGCLCs in response to the cytokine cocktail (Kinoshita et al., 2021). Interestingly, our germ cell-competent EpiSC models (E3 and T9) show relatively similar transcriptomic profiles to that of EpiLCs (Fig. S1E), compared with FSCs or EpiSCs without competence and cultured under the previous conditions (Kinoshita et al., 2021; Hayashi et al., 2011).

### LIF is not necessary for induction of PGCLCs from germ cell-competent EpiSCs

The induction of PGCLCs has been shown to require the stimulation of EpiLCs with a combination of BMP4 and leukemia inhibitory factor (LIF) (Hayashi et al., 2011), but the stimulation of *in vivo* epiblasts at E6.0 with only BMP4 (Ohinata et al., 2009). We thus examined whether LIF is required for the induction of PGCLCs from EpiSCs. As shown in Fig. 1C and Fig. S1F, PGCLCs were induced from the E3 and T9 lines stimulated with only BMP4, whereas PGCLC induction from EpiLCs was severely affected in the absence of LIF, consistent with previous research (Hayashi et al., 2011). Moreover, addition of stem cell factor (SCF) and epidermal growth factor (EGF) to the induction medium enhanced the induction efficiency of the stimulated E3 and T9 lines for the PGCLC population. Interestingly, culture conditions with these additional factors but without LIF exhibited greater effectiveness for PGCLC induction from the E3 and T9 lines than culture conditions with LIF, although PGCLC induction from EpiLCs was severely affected by the removal of LIF even in the presence of SCF and EGF.

Previous studies have suggested that high-dose exogenous BMP4 is required for induction of PGCLCs from *in vivo* epiblasts (Ohinata et al., 2009) and EpiLCs (Hayashi et al., 2011; Fig. S1H,I). We thus examined the dose-dependent effect of BMP4 on PGCLC induction from the E3 and T9 lines with various concentrations of BMP4 (Fig. 1D, Fig. S1G). Although E3 EpiSCs stimulated with 500 ng/ml of BMP4 exhibited the highest efficiency for PGCLC induction, the number of induced PGCLCs was similar to that for E3 EpiSCs treated with 250 ng/ml of BMP4. The induction efficiency of T9 EpiSCs was similar for the three different concentrations of BMP4 examined. Thus, culture conditions with 250 ng/ml of BMP4 but without LIF were used for the induction of PGCLCs from EpiSCs for further analyses.

### EpS-PGCLCs show characteristics similar to *in vivo* PGCs and EpL-PGCLCs

**Fig. 2.**
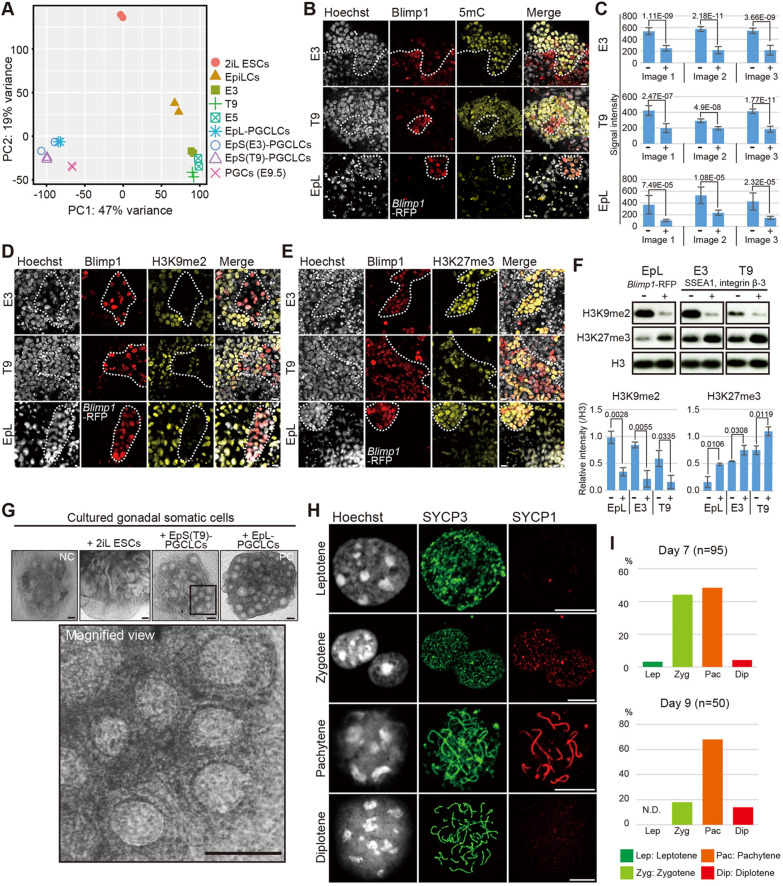
**Characterization of PGCLCs induced from EpiSCs.** (A) PCA of global gene expression in 2iL ESCs, EpiLCs, EpiSCs, PGCLCs and PGCs at E9.5. (B) Immunofluorescence analyses of 5 mC in PGCLCs induced from E3, T9 and EpiLCs (EpL) on day 6. Dotted lines delineate the boundaries of PGCLCs recognized by anti-BLIMP1 antibody staining (E3, T9) or *Blimp1*-RFP signal (EpL). Scale bars: 10 μm. (C) Analysis of signal intensity in 5 mC staining images. The 5 mC signal of ten BLIMP1-negative cells and ten BLIMP1-positive cells were measured by ImageJ. The signal intensity in three independent images of each cell line (E3, T9 and EpL) are shown ±s.d. *P*-values were calculated by two-tailed unpaired *t*-test. (D,E) Immunofluorescence analyses of H3K9me2 (D) and H3K27me3 (E) in PGCLCs induced from E3, T9 and EpL on day 6. Dotted lines delineate the boundaries of PGCLCs recognized by anti-BLIMP1 antibody staining (E3, T9) or *Blimp1*-RFP signal (EpL). Scale bars:10 μm. (F) Top: Western blot analysis of H3K9me2 and H3K27me3 in PGCLCs (*Blimp1*-RFP-positive cells or SSEA1 and integrin β-3 double-positive cells) and non-PGCLCs (*Blimp1*-RFP-negative cells or SSEA1- and integrin β-3-negative cells) in cultured EpL, E3 and T9 cell lines. Bottom: Quantification of H3K9me2 and H3K27me3 levels using H3 levels as a standard is shown ±s.d. calculated from three independent experiments. *P*-values were calculated by two-tailed unpaired *t*-test. (G) Brightfield images of representative reconstituted ovary (rOvary) on day 21. The top images show the cultured aggregate of gonadal somatic cells with no co-cultured cells, 2iL ESCs, EpS(T9)-PGCLCs and EpL-PGCLCs. NC, negative control; PC, positive control. The bottom image is a magnified view of the boxed area above. Scale bars: 100 μm. (H) Representative images of cultured EpS(T9)-PGCLCs in each stage of meiotic prophase I. Scale bars: 10 μm. (I) Quantification of cultured EpS(T9)-PGCLCs under meiotic progression. Percentages of each stage in total meiotic cells are shown. The meiotic cells in the rOvaries were obtained from two independent experiments at each stage.

To investigate the characteristics of EpS-PGCLCs, we first performed global gene expression analysis of EpiLCs, EpiSCs (E3, E5 and T9), and the induced PGCLCs from these epiblast models. RNA sequencing (RNA-Seq) analysis revealed that the PGCLCs induced from E3 and T9 EpiSCs bear similar transcriptomes to those of *in vivo* PGCs at E9.5 and EpL-PGCLCs (Fig. 2A).

Immunofluorescence analysis showed that 5 mC and H3K9me2 levels were downregulated and the H3K27me3 level was upregulated in EpS-PGCLCs (Fig. 2B-F), indicating that EpS-PGCLCs have a similar epigenetic state to EpL-PGCLCs and *in vivo* PGCs (Hayashi et al., 2011; Seki et al., 2005). These results indicate that the E3 and T9 EpiSC lines maintain a noticeable germ cell competence. Therefore, we hereafter refer to E3 and T9 EpiSCs as germ cell-competent EpiSCs (GC-EpiSCs).

We next examined the differentiation ability of EpS-PGCLCs. For this purpose, we used the established method for inducing primary oocytes from induced PGCLCs (Hikabe et al., 2016; Fig. S1K). Primary oocyte-like structures were observed in cultured reconstituted oocytes (rOvaries) containing PGCLCs (EpS-PGCLCs) induced from T9 (female EpiSCs) and gonadal somatic cells at E12.5, whereas these structures were not observed in cultured gonadal somatic cells without EpS-PGCLCs (Fig. 2G). Furthermore, immunofluorescence analysis for detection of the meiotic chromosome revealed the progression of meiotic prophase I from days 7 to 9 of culture (Fig. 2H,I), indicating that EpS-PGCLCs can enter and progress through meiosis.

### YAP is activated in GC-EpiSCs

**Fig. 3.**
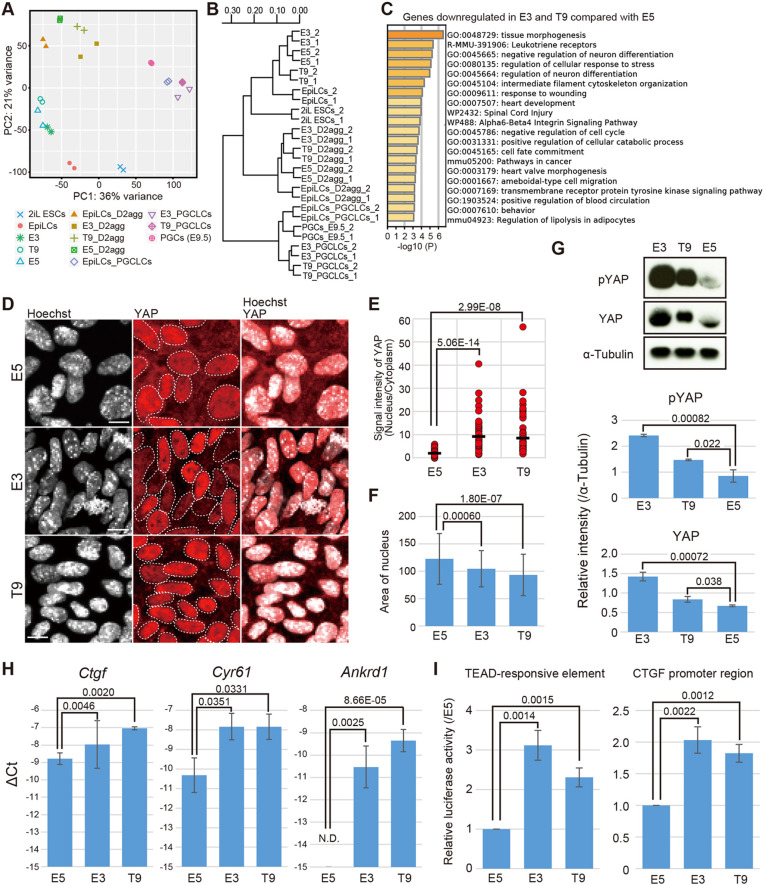
**YAP is activated in GC-EpiSCs.** (A,B) PCA (A) and clustering analysis (B) of global gene expression in induced PGCLCs at each developmental stage. The data for 2iL ESCs, EpiLCs, EpiSCs and PGCLCs are shown in Fig. 2A,B. (C) Functional enrichment analysis of genes downregulated in E3 and T9 compared with E5 cell lines. See also Table S1. (D) Immunofluorescence analysis of YAP in the E5, E3 and T9 cell lines. Scale bars: 10 μm. Dotted lines enclose nuclei. (E) Quantification of YAP signal in the immunostaining images of the E5, E3 and T9 cell lines (D). The signal intensity in the nucleus and cytoplasm of a total of 60 cells was measured by ImageJ in each cell line. Three independent experiments were performed (20 cells were analyzed in each individual experiment). The intensity of nucleus divided by the intensity of cytoplasm is shown. *P*-values were calculated by two-tailed unpaired *t*-test. (F) Area of the nucleus in the E5, E3 and T9 cell lines. The Hoechst-positive area of a total of 120 cells was calculated by ImageJ in each cell line. Three independent experiments were performed (40 cells were analyzed in each individual experiment). Average of the area is shown ±s.d. *P*-values were calculated by two-tailed unpaired *t*-test. (G) Top: Western blot analysis of phospho-YAP and YAP in the E3, T9, and E5 cell lines. Bottom: Quantification of phospho-YAP and YAP levels using α-tubulin levels as a standard is shown ±s.d. calculated from three independent experiments (ImageJ). *P*-values were calculated by two-tailed unpaired *t*-test. (H) The expression of *Ctgf*, *Cyr61* (*Ccn1*) and *Ankrd1* in the E5, E3 and T9 cell lines on day 2 after cytokine stimulation for PGCLC induction as measured by Q-PCR. The ΔCT from the average CT values of the two independent housekeeping genes, *Arbp* and *Ppia*, was calculated. The average value from three independent experiments (log_2_ scale) is shown ±s.d. *P*-values were calculated by two-tailed unpaired *t*-test. (I) TEAD-responsive element- and *Ctgf* promoter region-dependent luciferase reporter activity in the E5, E3 and T9 cell lines. Luciferase activities in E3 and T9 are shown relative to the activity in E5 as a standard (± s.d.). *P*-values were calculated by two-tailed unpaired *t*-test (three independent experiments with three technical replicants).

To gain insights into the mechanism underlying germline competence, we performed global gene expression analysis of GC-EpiSCs (E3 and T9), non-GC-EpiSCs (E5) and EpiSC-derived aggregates stimulated by cytokines. Principal component analysis (PCA) and unsupervised hierarchical clustering analysis revealed that GC-EpiSCs have a similar transcriptome to non-GC-EpiSCs, suggesting that maintenance of competence is not obviously reflected in the global gene expression profile of EpiSCs (Fig. 3A,B). Indeed, E3 EpiSCs with germline competence (GC) are more closely located to E5 EpiSCs without GC than to T9 GC-EpiSCs. Furthermore, EpiLCs with GCs clustered in a distinct branch away from the cluster of EpiSCs with or without GC, and a similar tendency was observed in cytokine-stimulated aggregates on day 2.

We then focused on the specific gene expression difference between GC-EpiSCs and non-GC-EpiSCs. This analysis identified 169 genes that were downregulated (Table S1) and 80 genes that were upregulated (Table S2) in GC-EpiSCs. Functional enrichment analysis showed that the terms related to ‘tissue morphology’, ‘cytoskeleton’ and ‘the response to stress’ are noticeably enriched in the genes downregulated in GC-EpiSCs (Fig. 3C). Of the regulatory mechanisms of these cellular processes, the Hippo signaling pathway has been established as crucial for these events (Dupont et al., 2011; Meng et al., 2016; Zhao et al., 2007). Although the function of BMP and WNT signaling has been determined in a germ cell differentiation context, it is unknown whether the Hippo signaling pathway is involved in this context. YAP and TAZ factors of the Hippo signaling pathway function as intracellular mediators for regulating gene expression. In the activated Hippo signaling context induced by cellular stress, such as cell-to-cell attachment, nucleus localization of these mediators is inhibited, leading to the inactivation of YAP/TAZ-dependent transcription. Of note, previous studies have shown that YAP or TAZ collaborates with SMADs and β-catenin, mediators of other signaling pathways, such as BMP and WNT signaling, to regulate its downstream targets (Alarcon et al., 2009; Azzolin et al., 2014; Piersma et al., 2015). Moreover, YAP knockout embryos show defects in extra-embryonic mesoderm (EXM), such as the allantois, where PGC specification is induced (Morin-Kensicki et al., 2006) at E7.5. A similar defect in EXM is also observed in mutant embryos in which a component of BMP and WNT signaling pathways (e.g. BMP4/SMAD1 and WNT3/β-catenin) has been deleted, which exhibit severely affected PGC specification (Aramaki et al., 2013; Lawson et al., 1999; Ohinata et al., 2009; Tremblay et al., 2001), suggesting that YAP mutant embryos may likewise exhibit a similar defect in PGC development. By contrast, TAZ knockout embryos show no defects in EXM and proceed through normal embryonic development (Hossain et al., 2007; Makita et al., 2008).

Our RNA-Seq analysis revealed that *Yap* is expressed in all EpiSC lines at similar levels as other highly activated genes, such as *Gapdh* and *Oct4* (*Pou5f1*) (Fig. S2D). In addition, previous studies have shown that there are two different spliced isoforms of YAP (Sudol et al., 1995). Our Q-PCR analysis revealed that the two different isoforms are noticeably activated at similar levels in all of the examined EpiSCs (Fig. S2E-G). Furthermore, no noticeable gene expression differences in major upstream components of Hippo signaling, such as *Mst1* and *Lats1/2*, were observed between GC- and non-GC-EpiSCs (Fig. S3A).

Interestingly, however, YAP protein was exclusively localized to the nuclei of GC-EpiSCs (E3 and T9), whereas cytoplasmic localization of YAP was clearly observed in non-GC-EpiSCs (E5) (Fig. 3D,E), suggesting that Hippo signaling is upregulated in non-GC-EpiSCs through the cellular events described above. In addition, GC-EpiSCs exhibited nuclei of smaller size than non-GC-EpiSCs (Fig. 3F), which might cause the distinct YAP localization according to different mechanical stress factors (Meng et al., 2016). Consistent with the immunofluorescence analysis, GC-EpiSCs exhibited higher expression of phosphorylated YAP and pan-YAP protein compared with non-GC-EpiSCs (Fig. 3G). Q-PCR and RNA-Seq analyses also revealed that GC-EpiSCs (E3 and T9) show higher expression of YAP target genes after cytokine stimulation compared with non-GC-EpiSCs (E5) (Fig. 3H, Fig. S3B). Furthermore, a luciferase reporter assay revealed that transcriptional activity mediated by the sequence of the TEAD-responsive element and the promoter sequence of *Ctgf* (*Ccn2*), respectively, is more highly activated in GC-EpiSCs compared with non-GC-EpiSCs (Fig. 3I). Thus, these findings collectively suggest that activated nuclear YAP may be involved in the establishment of EpiSC responsiveness to inductive signals or germline competence.

### YAP contributes to PGC specification

**Fig. 4.**
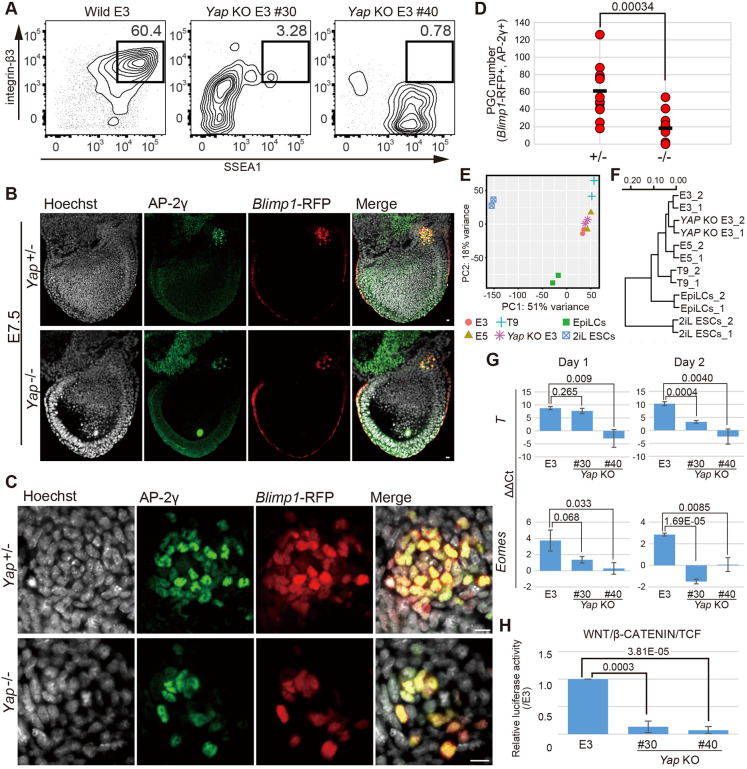
**YAP contributes to PGC induction through the WNT/β-catenin pathway.** (A) Representative FACS plots of SSEA1 and integrin β-3 expression in cultured aggregates derived from *Yap* KO E3 cells under conditions for inducing PGCLCs from EpiSCs (three independent experiments). The percentage of the induced PGCLC population is shown on each plot. See also Fig. S3D. (B,C) Immunofluorescence analysis of AP-2γ in *Yap*-mutant embryos containing the *Blimp1*-RFP transgene at E7.5. Whole epiblasts (B) and the region at the base of allantois (C) are shown. Scale bars: 10 μm. (D) The number of PGCs (*Blimp1*-RFP+, AP-2γ+) in *Yap*^+/−^ and *Yap*^−/−^ embryos at E7.5. Black bar shows average. *P*-values were calculated by two-tailed unpaired *t*-test. (E,F) PCA (E) and clustering analysis (F) of global gene expression in wild-type E3 and *Yap* KO E3 (line #40). The data for 2iL ESCs, EpiLCs and EpiSCs are shown in Figs 2A and 3A. (G) Gene expression dynamics of WNT target genes (*T*, *Eomes*) in the first 2 days of PGCLC induction from wild-type E3 and *Yap* KO E3 cell lines. For each gene examined, ΔΔCT was calculated from the ΔCT on day 0, which was calculated from the Ct value of the two independent housekeeping genes *Arbp* and *Ppia*. The average value from three independent experiments (log_2_ scale) is shown ±s.d. *P*-values were calculated by two-tailed unpaired *t*-test. (H) WNT/β-catenin/TCF-dependent luciferase reporter activity in wild-type E3 and *Yap* KO E3 cell lines. Luciferase activities in *Yap* KO E3 are shown relative to the activity in wild-type E3 as a standard (±s.d.). *P*-values were calculated by two-tailed unpaired *t*-test (three independent experiments with three technical replicants).

To investigate the function of YAP in the induction of PGCLCs from EpiSCs, we generated *Yap*-knockout E3 EpiSCs (*Yap* KO E3) with the CRISPR/Cas9 system (Fig. S3C) and induced PGCLCs from *Yap* KO E3. As shown in Fig. 4A and Fig. S3D, germ cell competence is severely affected in *Yap* KO E3. Although the expression of representative PGC determinants [*Blimp1* (*Prdm1*), *Prdm14* and *Tfap2c* (encoding AP-2γ)] was not clearly affected at early time points (days 0, 2 and 4) after cytokine stimulation, the expression *Blimp1* and *Prdm14* was affected at a later point, on day 6 (Fig. S3H), consistent with the fluorescence-activated cell sorting (FACS) analysis shown in Fig. 4A. We also established inducible *Yap* KO ESCs to explore the function of YAP in an EpiLC- or PGCLC-differentiation context (Fig. S3I). However, inducible deletion of *Yap* during EpiLC derivation from naïve ESCs led to massive cell death, precluding loss-of-function analysis (Fig. S3I).

We also examined the function of YAP in PGC development *in vivo*. There were fewer *Blimp1*-RFP and AP-2γ double-positive PGCs (Ohinata et al., 2005; Weber et al., 2010) in *Yap* knockout mutants (*Yap*^–/–^) than in heterozygous mutants (*Yap*^+/–^) (Fig. 4B-D). By contrast, no significant negative effect was observed in *Yap* heterozygous mutants (*Yap*^+/–^) (Fig. S3E-G).

To explore further the role of YAP in the PGCLC induction system, we performed RNA-Seq analysis of *Yap* KO E3. PCA and unsupervised hierarchical clustering of global gene expression, however, revealed that *Yap* KO E3 clustered together with wild-type E3 in the same distinct branch, suggesting that these cell lines have relatively similar transcriptomes, despite their distinct germ cell competence (Fig. 4E,F). This is consistent with the RNA-Seq result shown in Fig. 3A,B, indicating that the difference in germ cell competence between the EpiSC lines is not reflected in their global gene expression profile in the clustering analysis.

The WNT signaling pathway and its downstream targets (e.g. WNT3, β-catenin and T) are essential for the growth of mesoderm-derived tissues, including both somatic and germ cell lineages (Aramaki et al., 2013; Huelsken et al., 2000; Liu et al., 1999; Ohinata et al., 2009). Additionally, like mutants of such WNT signal components, *Yap* knockout embryos exhibited similar morphological defects in the mesoderm region (Morin-Kensicki et al., 2006). Therefore, we examined the response of *Yap* KO E3 to stimuli of PGCLC induction by quantifying the gene expression dynamics of WNT targets (Fig. 4G). WNT-related genes, such as *T* and *Eomes*, were found to be upregulated in wild-type E3 EpiSCs on day 2 after cytokine stimulation for PGCLC induction. However, in *Yap* KO E3 EpiSCs, the activation of these WNT target genes was noticeably affected. To confirm the disrupted WNT signaling pathway in *Yap* KO E3 lines, we analyzed the WNT/β-catenin/TCF activity by luciferase assay (Fig. 4H). Consistent with the affected activation of WNT target genes shown above, *Yap* KO E3 showed reduced WNT/β-catenin/TCF activity compared with wild-type E3 on day 2 after cytokine stimulation for PGCLC induction.

### Exogenous *Yap* overexpression in non-GC-EpiSCs restores competence

**Fig. 5.**
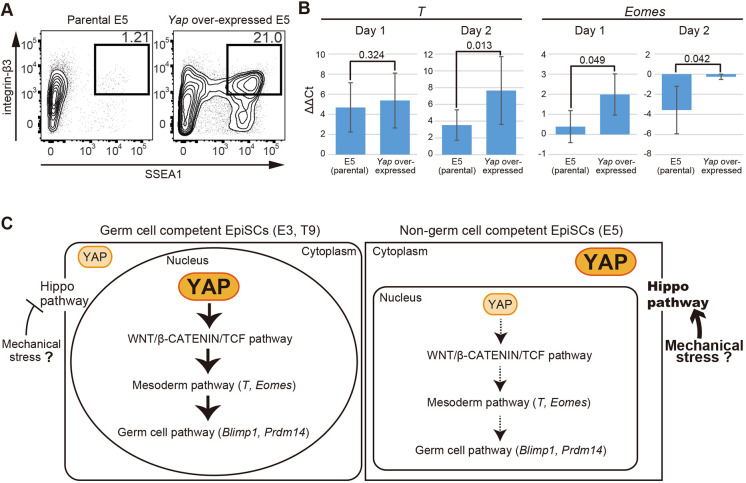
**Exogenous *Yap* overexpression restores PGC competence in non-GC-EpiSCs.** (A) Representative FACS plots of SSEA1 and integrin β-3 expression in cultured aggregates derived from parental E5 and *Yap* overexpressing E5 cells under conditions for inducing PGCLCs from EpiSCs (two independent experiments). The percentage of the induced PGCLC population is shown on each plot. See also Fig. S4C,D. (B) Gene expression dynamics of WNT target genes (*T*, *Eomes*) in the first 2 days of PGCLC induction from parental E5 and *Yap* overexpressing E5 cells. For each gene examined, ΔΔCT was calculated from the ΔCT on day 0, which was calculated from the Ct value of the two independent housekeeping genes *Arbp* and *Ppia*. The average value from three independent experiments (log_2_ scale) is shown ±s.d. *P-*values were calculated by two-tailed unpaired *t*-test. (C) Model indicating that YAP creates epiblast germ cell competence through the establishment of responsiveness to cytokine stimulation for activation of WNT target genes and subsequent induction of germ cell fate.

Finally, to examine whether activation of *Yap* restores the germ cell differentiation ability of incompetent EpiSCs, exogenous *Yap* was overexpressed in non-GC-E5 EpiSCs (Fig. S4A,B) by using the Tet-on system. FACS analysis revealed that PGCLCs are induced from *Yap*-overexpressing E5 EpiSCs in response to stimulation with doxycycline, indicating that competence is restored by exogenous *Yap* activation (Fig. 5A, Fig. S4C,D).

Furthermore, we found that *Yap* overexpression also restores the activation of WNT target genes, such as *T* and *Eomes*, at an early time point after cytokine stimulation (days 1 and 2) (Fig. 5B), consistent with the loss-of-function analysis described above (Fig. 4). As T is one of the WNT targets that directly regulates PGC determinants, such as Blimp1, according to T dosage and its related pluripotency (Aramaki et al., 2013, 2021), these results suggest that the activation competence of WNT targets for germ cell fate induction is established by YAP (Fig. 5C).

## DISCUSSION

Although the induction of functional PGCLCs *in vitro* is a robust system for investigating the molecular mechanism of PGC specification (Hayashi et al., 2011), it is still unclear how the differentiation ability of epiblast cells into the germ cell lineage is established and maintained. A previous study has suggested that the *in vivo* epiblast has a specific time window for responding to external cues for germ cell induction (Ohinata et al., 2009). In addition, EpiLCs, a transient state of differentiating ESCs but not self-renewing EpiSCs, have competence for germline induction (Hayashi et al., 2011). However, the original study by Tesar et al. showed that the T9 line has responsiveness to cytokine stimuli for PGC specification, suggesting that the GC of EpiSCs is not well defined. Also, previous studies have shown that different EpiSC lines exhibit distinct features of pluripotency and differentiation ability (Bernemann et al., 2011; Song et al., 2016). Thus, using the previously established lines representing early post-implantation epiblast, we here examined the PGCLC induction ability of three different EpiSC lines: E3, E5 and T9. Contrary to the previous report (Hayashi et al., 2011), we determined the competence for PGCLC induction of two distinct EpiSC lines, E3 and T9 (Fig. 1A), and then explored the mechanism for the establishment and maintenance of germ cell competence. Our findings here suggest that undefined factors in MEF-derived condition medium are crucial for maintaining competence. We consider that the observed variability in the derivation efficiency of PGCLCs from EpiSCs may have to do with the different batches of CM used (Fig. S1J).

From these examined lines, PGCLCs were induced by only BMP4 in the absence of LIF (Fig. 1C, Fig. S1F), suggesting similarity between GC-EpiSCs and *in vivo* epiblasts with GC (Ohinata et al., 2009). As LIF is required for the maintenance of ESC pluripotency, this result suggests that GC-EpiSCs have a marked ability for retaining pluripotency during the induction of PGCLCs. Indeed, representative pluripotency-related genes such as *Oct4* and *Sox2* were found to be more highly maintained in GC-EpiSCs, compared with non-GC-EpiSCs, and were annotated to the terms such as ‘mechanisms associated with pluripotency’ and ‘cellular response to leukemia inhibitory factor’ (Fig. S2B,C, Tables S3 and S4).

The Hippo/YAP pathway has been shown to be involved in the proliferation and differentiation of murine ovarian germline stem cells (Ye et al., 2017). However, its function at the onset of germ cell specification from pluripotent epiblast was unknown. Our RNA-Seq analysis suggests that the activity of the Hippo pathway is downregulated in GC-EpiSCs, compared with non-GC-EpiSCs. In line with the RNA-Seq result, YAP is localized exclusively to the nuclei of GC-EpiSCs. In contrast, YAP is clearly observed in the cytoplasm of non-GC-EpiSCs, which exhibited a larger nuclear size compared with GC-EpiSCs in the immunofluorescence images (Fig. 3D-F), suggesting activation of the Hippo signaling pathway. Moreover, we found that *Yap* KO E3 and *Yap* knockout embryos show a defect in germ cell induction (Fig. 4A-D). Like *Yap* KO embryos, *Yap* KO EpiSCs showed poor induction of germ cells (Fig. 4A). We found that expression of PGC determinants, such as *Blimp1* and *Prdm14*, are not affected at early time points (days 0, 2 and 4) after cytokine stimulation (Fig. S3H). In this context, TAZ, another Hippo downstream factor, is stably activated. Thus, we suggest that TAZ, or other factors, compensate for the loss of YAP function. Although the transcriptome of *Yap* KO E3 EpiSCs is similar to that of wild-type E3 EpiSCs, the responsiveness of *Yap* KO E3 to cytokines, estimated by the activation of WNT target genes (*T* and *Eomes*) and WNT/β-catenin/TCF transcriptional activity, was severely affected (Fig. 4E-H).

The induction ability of WNT target genes that are crucial for PGC specification and the consequent generation of PGCLCs were rescued by exogenous *Yap* in incompetent E5 EpiSCs. Future investigations may provide additional information on the molecular characterization and the relevant underlying mechanism.

Previous studies have suggested that the differentiation program orchestrated by mesodermal transcription factors is an unnecessary by-product of nascent gastrulation as a result of its eventual inactivation (Kurimoto et al., 2008; Ohinata et al., 2005). However, more recent studies have clearly indicated that pan-mesodermal transcription factors activated by upstream factors such as WNT signaling are required for the onset of germ cell fate induction (Aramaki et al., 2013; Chen et al., 2017; Kojima et al., 2017). Indeed, the well-defined WNT target T (brachyury) is required for germ cell specification and regulates germ cell determinant genes (Aramaki et al., 2013). A very recent study has shown that T directly activates the program of germ cell segregation from somatic lineages according to T dosage and its related residual pluripotency (Aramaki et al., 2021). Therefore, our findings here suggest that the establishment of germ cell competence or the responsiveness of epiblast stem cell lines to cytokines for activating WNT signaling is created by YAP, whereas the exact mechanism of germline segregation from the somatic mesoderm presumably regulated by the same YAP/WNT pathway remains unclear.

In conclusion, we have shown here, for the first time, that different EpiSC lines exhibit distinct germ cell competence. The robust induction of PGCLCs from GC-EpiSCs suggests that EpiSCs serve as a useful source for investigating the regulatory mechanisms for germ cell differentiation. Using this model, we identified that YAP, a mediator of the Hippo signaling pathway, is required for the establishment of responsiveness to inductive stimuli for the specification of the germ cell lineage.

## MATERIALS AND METHODS

### Mice

All mice used in this study were bred and housed in the animal facility of the Max Planck Institute (MPI) in Muenster. Female mice were housed in groups of up to five per cage and male mice were housed singly in individually ventilated, type II long cages under a 14-h light/10-h dark cycle with a controlled temperature of 22°C, 40-60% humidity, and with free access to water and rodent chow. Procedures used followed the ethical and experimental recommendations of the German Society of Laboratory Animal Science. At the local regulatory level, mice were used for experiments according to the ethical approval issued by the Landesamt für Natur, Umwelt und Verbraucherschutz of the state of North Rhine-Westphalia, Germany (permit nos: 84-02.04.2016.A015, 81-02.04.2017.A376, and 81-02.04.2017.A493).

For genetic experiments, Zp3-Cre transgenic female mice (de Vries et al., 2000) were interbred with loxP-flanked *Yap* (*Yap*^lox/lox^)-carrying male mice (Reginensi et al., 2013). *Blimp1*-RFP transgenic mice (Sugimoto and Abe, 2007) were used to monitor primordial germ cells in *Yap* mutant embryos. To obtain *Yap*^−/−^ or *Yap*^−/+^ *Blimp1*-RFP transgenic embryos in the same litter, *Yap*^−/+^ *Blimp1*-RFP transgenic male mice were interbred with *Yap*^lox/lox^ Zp3-Cre *Blimp1*-RFP transgenic female mice. To obtain *Yap*^−/+^ or *Yap*^+/+^ embryos in the same litter, *Yap*^−/+^ *Blimp1*-RFP transgenic male mice were interbred with CD1 female mice.

### Cell culture

E3 male EpiSCs, E5 male EpiSCs [GOF18-GFP (C57BL/6 and DBA/2)×129Sv] (Greber et al., 2010; Yoshimizu et al., 1999) and T9 female EpiSCs (129SvEv or 129S2/SvHsd) (Tesar et al., 2007) were maintained as previously described (Greber et al., 2010) in MEF-CM (Xu et al., 2001) containing 10 ng/ml of FGF2 (CMF) on dishes coated with fetal calf serum (FCS). Fresh CMF was added to EpiSCs every day and cells were passaged at a 1:20 ratio every 3 days. The details of the derivation of *Oct4*/GOF18ΔPE-EGFP, *Blimp1*-RFP (BR) male ESCs and *Yap*^−/lox^, *Blimp1*-RFP male ESCs have been previously described (Aramaki et al., 2021). ESCs were cultured in N2B27 medium with 2i (1 μM PD0325901 and 3 μM CHIR99021) plus LIF (Ying et al., 2008) on dishes coated with 0.01% poly-L-ornithine (Sigma-Aldrich) and laminin (10 ng/ml; BD Biosciences) under feeder-free conditions. To establish the doxycycline- and tamoxifen-inducible *Yap* knockout ESC line, the pPBCAG-CreERT2 vector harboring a tetracycline responsible element was transfected into *Yap*^−/lox^, *Blimp1*-RFP male ESCs using Lipofectamine 2000 with a pPyCAG-PBase vector and a pPBCAG-rtTA-IRES-Neo vector harboring a neomycin resistance gene. After 7 days of neomycin/G418 selection (250 μg/ml; Life Technologies), single clones were used for experiments.

For culturing E3 and T9 in N2B27 medium containing activin A (20 ng/ml), bFGF (12 ng/ml) and KSR (20%), the dishes were coated with human plasma fibronectin (16.7 μg/ml), the medium was changed every day, and cells were passaged at a 1:20 ratio every 2-3 days.

### PGCLC induction

EpL-PGCLCs were induced as described previously (Hayashi et al., 2011). Briefly, 5×10^4^ ESCs were cultured per well in 24-well plates coated with human plasma fibronectin (16.7 μg/ml) in N2B27 medium containing activin A (20 ng/ml), bFGF (12 ng/ml) and KSR (1%). After 24 h, the medium was changed, and the cells were cultured for another 24 h. The resultant EpiLCs were then cultured under floating conditions by plating 2×10^3^ cells per well of Ultra Low Cluster round-bottom 96-well plates (Costar) in GK15 medium in the presence of cytokines BMP4 (500 ng/ml), SCF (100 ng/ml), EGF (50 ng/ml) and LIF (1000 µ/ml).

To induce EpS-PGCLCs, the maintained EpiSCs were passaged at a 1:10 ratio per well of 12-well plates coated with FCS 2 days before the induction and then cultured under floating conditions by plating 2×10^3^ cells per well of Ultra Low Cluster round-bottom 96-well plates (Costar) in GK15 medium in the presence of the cytokines BMP4 (250 ng/ml), SCF (100 ng/ml), EGF (50 ng/ml) and ROCK inhibitor Y27632 (10 μM), which is added to improve the cell viability and the survival rate after trypsinization (Watanabe et al., 2007).

SSEA1 and integrin β-3 double-positive cells were sorted as PGCLCs from cultured E3 and T9 EpiSCs, as the SSEA1/integrin β-3 double-positive cells are the population recognized as being equivalent to *Blimp1* reporter-positive PGCLCs (Hayashi et al., 2011).

To induce PGCLCs from E5 with an inducible *Yap* overexpression system, doxycycline (1 μg/ml) was added to CM when cells were passaged 2 days before the induction. See also Fig. S4A.

### FACS analysis

PGCLCs were dissociated with 0.25% Trypsin-EDTA (Gibco), washed with an equal volume of EpiSC medium containing 20% Knockout Serum Replacement (Invitrogen), and then collected by centrifugation at 300 ***g*** for 5 min. The dissociated cells were incubated with anti-integrin β-3 antibody (BioLegend) and anti-SSEA1 antibody (R&D Systems) conjugated with Alexa Fluor 647 and phycoerythrin, respectively, in PBS containing 0.1% bovine serum albumin (BSA). After washing with PBS containing 0.1% BSA, the cells were filtered through a cell strainer (70 μm; BD Biosciences) and then sorted and analyzed on a flow cytometer (ARIA III; BD Biosciences). FACS plots were created using Flowjo.

### Q-PCR and RT-PCR analysis

Total RNA was isolated using the NucleoSpin RNA Mini kit (MACHEREY-NAGEL) and was reverse transcribed with oligo-dT primer by M-MLV Reverse Transcriptase (USB; Affymetrix). Real-time PCR was performed using the iTaq Universal SYBR Green Supermix (Bio-Rad Laboratories) on the QuantStudio 5 Real-Time PCR System. Gene expression was normalized to the housekeeping genes *Arbp* (*Rplp0*) and *Ppia* and calculated using the ΔCt algorithm. More specifically, the ΔCt values from the average Ct values of *Arbp* and *Ppia* were calculated (log_2_ scale, the mean value of three independent experiments with two technical replicates). The primer sequences used are listed in Table S6.

To detect the *Yap* isoforms 1 and 2, total RNA was reverse transcribed with a random hexamer followed by quantified real-time PCR and amplification of the region surrounding the splicing variant by PCR with the following primers: 5′-CTCCAGTGAAGCAGCCCC-3′ and 5′-TGGTTGTCATTGTTCTCAATTCCTG-3′.

### Chromosome spreading

Preparation of the chromosome spreads was performed as described previously (Hikabe et al., 2016) with slight modifications. Reconstituted ovaries were dissociated by incubation with CTK (0.1 mg ml^−1^ collagenase IV, 0.25% Trypsin, 20% KSR and 1 mM CaCl_2_ in PBS) for 30 min at 37°C, followed by Accutase for 5 min at 37°C. Single dissociated cells were resuspended in hypotonic buffer [30 mM Tris-HCl (pH 8.2), 50 mM sucrose, 17 mM trisodium citrate dihydrate, 5 mM ethylenediaminetetraacetic acid (EDTA), 0.5 mM dithiothreitol (DTT), 0.1 mM phenylmethylsulfonyl fluoride (PMSF)] for 10 min at room temperature (RT) and placed onto glass slides that had been dipped in fixative solution [1% paraformaldehyde (PFA), 0.15% Triton X-100 in deuterium-depleted water (DDW) (pH 9.0)]. Cell spreads on the glass slides were incubated for 2 h at RT, washed in PBS, and then used for immunostaining.

### Immunofluorescence analysis

For immunostaining of H3K9me2 and H3K27me3, the cell aggregates were fixed with 4% PFA in PBS for 1 h on ice, washed with PBS, and incubated in PBS containing 0.1% BSA and 0.1% Triton X-100 for 30 min at 4°C for blocking and permeabilization. The cells were incubated with the primary antibody at 4°C for 4 days, and then incubated with the secondary antibody at 4°C for 2 days. For 5 mC immunostaining, the permeabilized cell aggregates were soaked in 4 N HCl, 0.1% Triton X-100 in DDW for 10 min for depurination, washed, and then incubated for blocking. Samples were incubated with the primary and the secondary antibodies at 4°C for 4 and 2 days, respectively. For SYCP3 and SYCP1 immunostaining, the cell spreads were incubated in a blocking solution of PBS containing 5% BSA for 1 h at RT and incubated with the primary antibody overnight at 4°C and then incubated with the secondary antibody and Hoechst for 1 h at RT. For YAP, EpiSCs cultured on glass-bottom dishes coated with FCS were fixed with 4% PFA in PBS for 15 min on ice, washed in PBS, followed by blocking and permeabilization in PBS containing 0.1% BSA and 0.1% Triton X-100 for 15 min at RT. The cells were incubated with the primary antibody at RT for 1 h, and then incubated with the secondary antibody at RT for 30 min. For AP-2γ immunostaining, E7.5 embryos were fixed in 4% PFA in PBS for 3 h on ice, washed in PBS, and then incubated for blocking and permeabilization in PBS containing 0.1% BSA and 0.1% Triton X-100 for 30 min at 4°C. They were incubated with the primary antibody at 4°C for 4 days, and then incubated with the secondary antibody at 4°C for 2 days. The primary antibodies used in this study were: anti-5 mC (1:100; AMM 99021, Aviva), anti-H3K9me2 (1:500; 07-441, Millipore), anti-H3K27me3 (1:500; 07-449, Millipore), anti-BLIMP1 (1:100; sc-47732, Santa Cruz Biotechnology), anti-SYCP3 (1:100; ab97672, Abcam), anti-SYCP1 (1:500; NB300-229, Novus Biologicals), anti-YAP (1:100; 14074, Cell Signaling Technology), anti-E-cadherin (1:100; ab76055, Abcam) and anti-AP-2γ (1:1000; sc-8977, Santa Cruz Biotechnology). The secondary antibodies used in this study were: Alexa Fluor 488 anti-mouse or rat IgG, Alexa Fluor 568 anti-mouse or rabbit IgG, and Alexa Fluor 647 anti-mouse or rabbit IgG (all goat polyclonal; A28175, A11006, A11004, A11011, A21235 and A21245, Invitrogen). The images were captured using a confocal microscope (Zeiss LSM780).

For quantification of immunostaining data, the signal intensity of 5 mC and YAP was quantified using ImageJ software. The signal intensity of 5 mC in the same size area was compared between BLIMP1^+^ cells and BLIMP1^−^ cells. The signal intensity of YAP in an area of the same size was compared between the cytoplasm and nucleus of each cell. For quantitative analysis, three independent experiments were performed, and in each individual experiment 20 cells were analyzed. *P*-values were calculated by two-tailed unpaired *t*-test.

### RNA-Seq

Total RNA was extracted from each cell population using the NucleoSpin RNA Mini kit (MACHEREY-NAGEL). Then, secondary RNA purification was performed using the NEB Next Poly A m-RNA magnetic bead selection kit (E7490), and each cDNA library was prepared using the NEBNext Ultra II RNA Library Prep Kit for Illumina (E7770). Sequencing was performed using the Illumina Nextseq 500 platform with paired-end 75-bp reads. Sequenced reads were aligned to the mm10 reference genome using Bowtie2 (Langmead and Salzberg, 2012) with default parameters. Rsubread R package (Liao et al., 2019) was used to count the number of RNA-Seq reads. Screening of the differentially expressed genes, clustering analysis, and PCA analysis were performed using DESeq2 (Love et al., 2014) on the iDEP9.1 platform (Ge et al., 2018). Functional enrichment analysis was performed on Metascape (Mizukami et al., 2016; Zhou et al., 2019).

The RNA-Seq datasets of FSCs or EpiSCs cultured in N2B27-based medium in the previous study (Kinoshita et al., 2021) were used for PCA analysis.

### Generation of mutant EpiSCs with the CRISPR/Cas9

Mutant EpiSC lines were generated with the CRISPR/Cas9 system (Cong et al., 2013; Mali et al., 2013; Slaymaker et al., 2016). sgRNA oligonucleotides were subcloned downstream of the human U6 promoter of pX330A-GFPT2APuro (modified from Addgene #42230, deposited by Feng Zhang) (Adachi et al., 2018). To generate YAP KO EpiSCs, two sgRNAs were designed to delete the first exons, leading to frameshift mutations. The constructed plasmids were transfected into cells using Lipofectamine 2000, followed by selection with puromycin (1 μg/ml) for 2 days. Selected clones bearing homozygous deletions were subjected to sequencing of the genomic PCR products. Sequences of the sgRNA oligonucleotide used in this study are listed in Table S5.

### Establishment of E5 cell lines with the Tet-on inducible YAP overexpression system

For vector construction, cDNA encoding 3xFlag-tagged *Yap* was amplified by PCR and inserted into pPB-hCMV1-pA vector harboring a tetracycline responsible element. Using Lipofectamine 2000, the constructed vector above was transfected into E5 together with the pPBCAG-rtTA-IRES-Neo vector harboring a neomycin resistance gene. To avoid excessive exogenous *Yap* expression, a pPyCAG-PBase was not co-transfected. After 7 days of neomycin/G418 selection (250 μg/ml; Life Technologies), single clones were used for experiments.

### Western blot analysis

For western blot analysis of H3K9me2 and H3K27me3 (Fig. 2F), 1×10^4^ cells from each sample were lysed in SDS sample buffer [62.5 mM Tris-HCl (pH 6.8), 2% sodium dodecyl sulfate (SDS), 10% glycerol, 0.025% Bromophenol Blue and 0.14 M β-mercaptoethanol] at 95°C for 5 min. Proteins were separated on 15% acrylamide gel, blotted onto Immobilon-P transfer membrane (Millipore), and incubated with the primary antibodies [anti-H3K9me2 (1:5000; 07-441, Millipore), anti-H3K27me3 (1:1000; 07-449, Millipore), anti-H3 (1:20,000; ab1791, Abcam)]. Primary antibodies were detected with the secondary antibodies conjugated with HRP (111-035-144, Jackson ImmunoResearch), followed by detection using ECL Prime (GE Healthcare).

For western blot analysis of pYAP and YAP (Fig. 3G), 2×10^4^ cells from each sample were lysed, separated on 10% acrylamide gel, and blotted as described above. The membrane was incubated with primary antibodies [anti-pYAP (1:250; 4911, Cell Signaling Technology), anti-YAP (1:1000; 4912, Cell Signaling Technology), anti-α-tubulin (1:20,000; T6199, Sigma-Aldrich)], followed by detection.

For western blot analysis of YAP (Fig. S3B), 5×10^4^ cells from each sample were lysed, separated on 10% acrylamide gel, and blotted as described above. The membrane was incubated with primary antibodies [anti-YAP (1:1000; 4912S, Cell Signaling Technology), anti-α-tubulin (1:20,000; T6199, Sigma-Aldrich)], followed by detection.

### Luciferase reporter assay

The DNA sequences of the 8xTEAD binding element, the *Ctgf* promoter region (Xu et al., 2015) and the WNT/β-catenin/TCF responsive element were subcloned into a PiggyBAC-based firefly luciferase reporter plasmid upstream of a minimal TK promoter, respectively. The constructed reporter vector (40 ng/well) was transfected together with *Renilla* Luciferase Control Reporter Vector (4 ng/well) into EpiSCs using Lipofectamine 2000 (Invitrogen) according to the manufacturer's protocol. Transfected cells were lysed at 48 h post-transfection for reporter analyses using the Dual-Glo Luciferase Assay System (Promega). The relative luciferase reporter activity is shown with s.d. (three independent experiments with three technical replicates).

## Supplementary Material

Supplementary information

Reviewer comments
