# Supplementary material for: YAP establishes epiblast responsiveness to inductive signals for germ cell fate
Source: Development. 2021 Oct 19;148(20):dev199732. doi: 10.1242/dev.199732 (PMC8571999; doi:10.1242/dev.199732)
Supplement: Supplementary information [file develop-148-199732-s1.pdf]

A

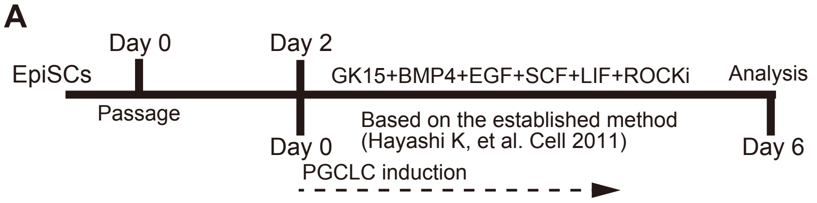

B

	EpiLCs	E3	E5	T9
1st	6.57%	74.90%	4.23%	93.20%
2nd	3.70%	29.60%	3.50%	33.10%
3rd	4.70%	33.70%	3.00%	32.30%

C

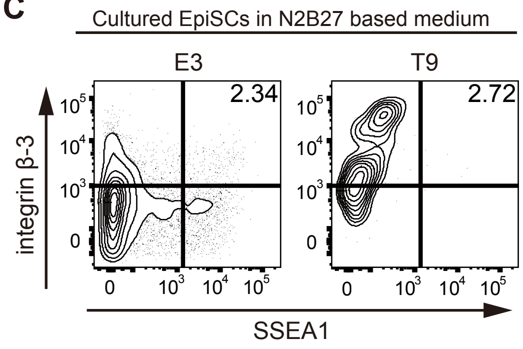

E

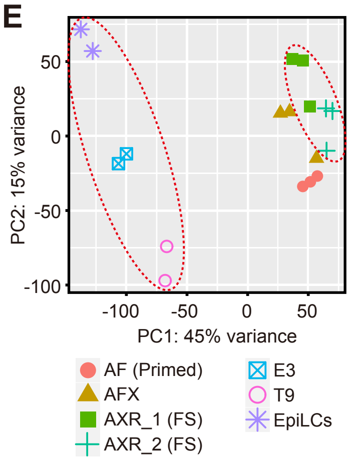

F

1st

	EpiLCs	E3	T9
BMP4	0.073%	7.47%	21.6%
BMP4 +SCF+EGF	0.28%	46.0%	42.6%
BMP4+LIF +SCF+EGF	2.88%	33.5%	32.1%

2nd

	EpiLCs	E3	T9
BMP4	0.100%	6.94%	15.8%
BMP4 +SCF+EGF	0.90%	64.0%	37.7%
BMP4+LIF +SCF+EGF	3.70%	56.7%	35.4%

D

	E3	T9
1st	2.34%	2.72%
2nd	3.28%	2.90%

H

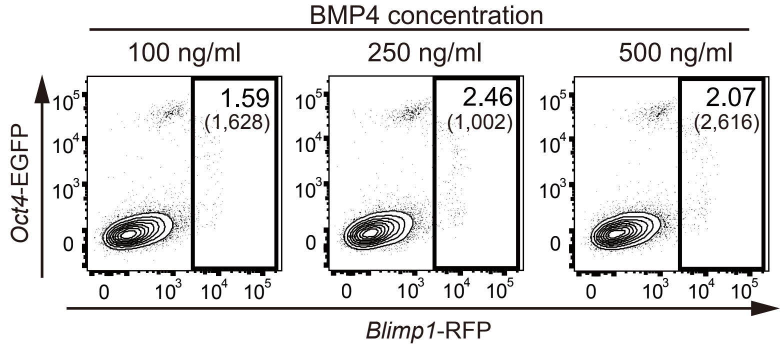

G

1st

	BMP4 concentration (ng/ml)		
	100	250	500
E3	8.06% (33,475)	14.5% (61,240)	18.9% (66,814)
T9	44.4% (32,715)	41.0% (24,181)	37.0% (35,602)

2nd

	BMP4 concentration (ng/ml)		
	100	250	500
E3	7.10% (7,761)	5.00% (18,565)	4.40% (14,312)
T9	57.0% (18,892)	56.5% (20,426)	50.9% (29,214)

I

	BMP4 concentration (ng/ml)		
	100	250	500
1st	1.59% (1,628)	2.46% (1,002)	2.07% (2,616)
2nd	3.00% (1,339)	1.90% (1,008)	2.80% (1,143)

J

	E3	T9
Lot_22.11.2015	73.0%	93.0%
(Lot_19.01.2016	4.20%	13.8%)
Lot_14.02.2018	35.4%	68.6%
Lot_09.03.2021	29.7%	56.5%

K

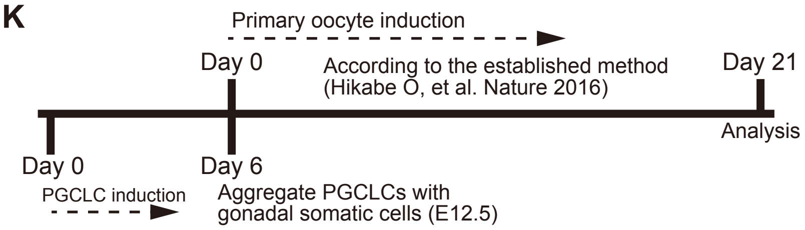

Development • Supplementary information

Fig. S1. PGCLC induction from EpiSCs and EpiLCs.

A) Scheme for primordial germ cell–like cell (PGCLC) induction from EpiSCs based on the established method (Hayashi et al., 2011).

B) The PGCLC induction rates analyzed by FACS in Fig. 1A (three independent experiments).

C) Representative FACS plot images of SSEA1 and integrin β-3 expression in the cultured aggregates. PGCLCs was derived from E3 and T9 cells cultured in N2B27-based medium as indicated by Hayashi et al. (two independent experiments). The percentage of the induced PGCLC population is shown. See also Fig. S1D.

D) The PGCLC induction rates analyzed by FACS in Fig. S1C (two independent experiments).

E) PCA analysis of global gene expression of GC-EpiSCs cultured in MEF-conditioned medium; EpiLCs; EpiSCs cultured in N2B27-based medium containing activin A and bFGF (AF); EpiSCs cultured in N2B27-based medium containing activin A, bFGF, and XAV939 (AFX); and EpiSCs cultured in N2B27-based medium containing activin A, XAV939, and BMS493 (AXR: the culture condition of germ cell–competent formative stem cells [FS cells]) (Kinoshita et al., 2021). Red circle encloses epiblast models harboring germ cell competence. The EpiSC culture condition of AF is also used in Hayashi et al., 2011.

F) The PGCLC induction rates analyzed by FACS in Fig. 1C (two independent experiments).

G) The PGCLC induction rates analyzed by FACS in Fig. 1D (two independent experiments).

H) Representative FACS plot images of *Blimp1*-RFP and *Oct4*/GOF18ΔPE-EGFP (*Oct4*-EGFP) in the cultured aggregates derived from EpiLCs induced from ESCs (*Blimp1*-RFP, *Oct4*/GOF18ΔPE-EGFP; BROG) under the indicated BMP4 concentration on day 6 (two independent experiments). The concentration of BMP4 is shown. The percentage and the number of the induced PGCLCs are shown. See also Fig. S1I.

I) The PGCLC induction rates analyzed by FACS in Fig. S1H (two independent experiments).

J) The PGCLC induction rates of E3 and T9 cultured in the different Lot-CM analyzed by FACS. Lot_19.01.2016 was not used for any experiments because of very low induction efficiency.

K) Scheme for primary oocyte induction from T9 cells according to the established method (Hikabe et al., 2016).

Development • Supplementary information

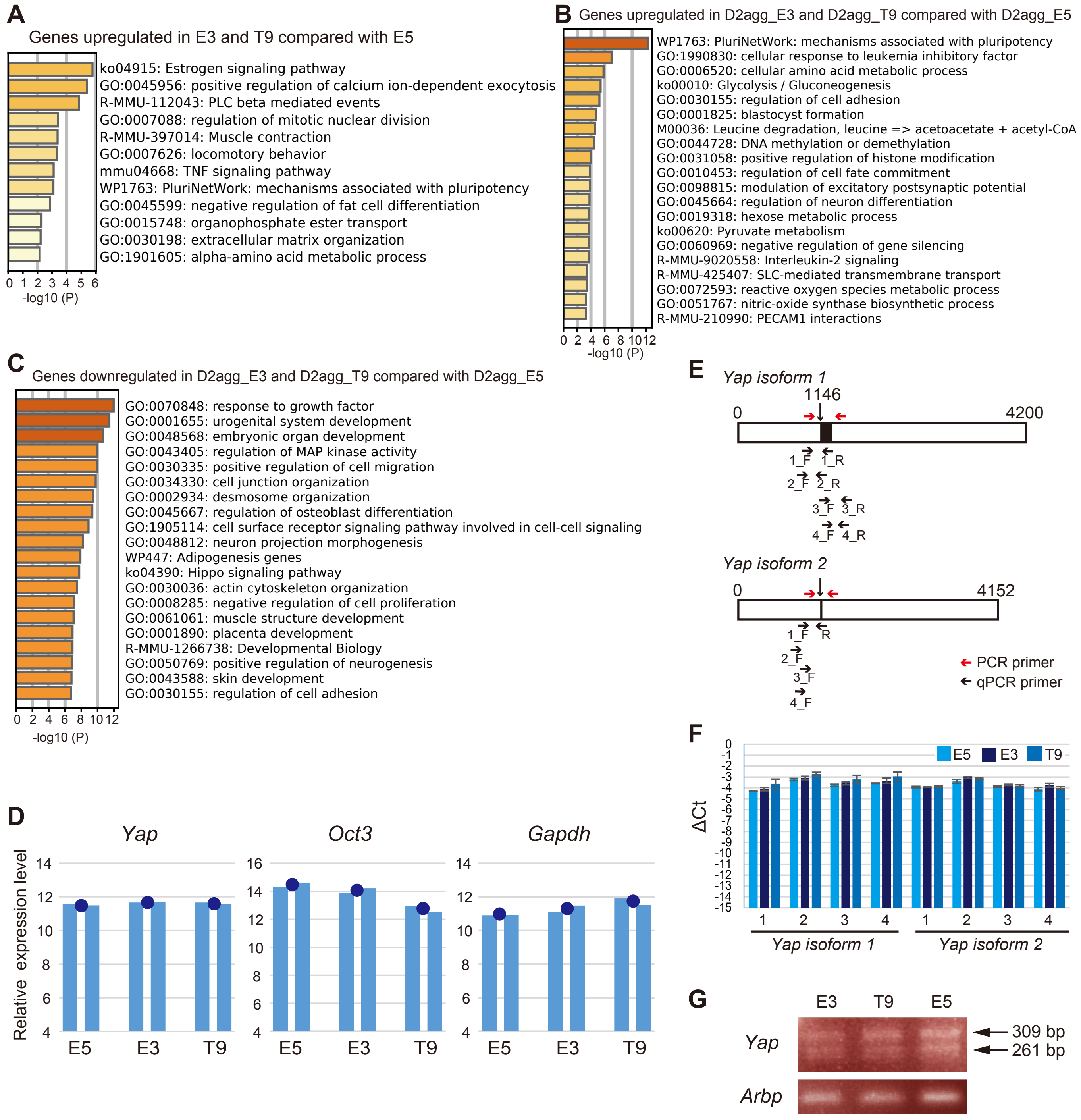
A
Genes upregulated in E3 and T9 compared with E5
ko04915: Estrogen signaling pathway
GO:0045956: positive regulation of calcium ion-dependent exocytosis
R-MMU-112043: PLC beta mediated events
GO:0007088: regulation of mitotic nuclear division
R-MMU-397014: Muscle contraction
GO:0007626: locomotory behavior
mmu04668: TNF signaling pathway
WP1763: PluriNetWork: mechanisms associated with pluripotency
GO:0045599: negative regulation of fat cell differentiation
GO:0015748: organophosphate ester transport
GO:0030198: extracellular matrix organization
GO:1901605: alpha-amino acid metabolic process
-log10 (P)
B
Genes upregulated in D2agg_E3 and D2agg_T9 compared with D2agg_E5
WP1763: PluriNetWork: mechanisms associated with pluripotency
GO:1990830: cellular response to leukemia inhibitory factor
GO:0006520: cellular amino acid metabolic process
ko00010: Glycolysis / Gluconeogenesis
GO:0030155: regulation of cell adhesion
GO:0001825: blastocyst formation
M00036: Leucine degradation, leucine => acetoacetate + acetyl-CoA
GO:0044728: DNA methylation or demethylation
GO:0031058: positive regulation of histone modification
GO:0010453: regulation of cell fate commitment
GO:0098815: modulation of excitatory postsynaptic potential
GO:0045664: regulation of neuron differentiation
GO:0019318: hexose metabolic process
ko00620: Pyruvate metabolism
GO:0060969: negative regulation of gene silencing
R-MMU-9020558: Interleukin-2 signaling
R-MMU-425407: SLC-mediated transmembrane transport
GO:0072593: reactive oxygen species metabolic process
GO:0051767: nitric-oxide synthase biosynthetic process
R-MMU-210990: PECAM1 interactions
-log10 (P)
C
Genes downregulated in D2agg_E3 and D2agg_T9 compared with D2agg_E5
GO:0070848: response to growth factor
GO:0001655: urogenital system development
GO:0048568: embryonic organ development
GO:0043405: regulation of MAP kinase activity
GO:0030335: positive regulation of cell migration
GO:0034330: cell junction organization
GO:0002934: desmosome organization
GO:0045667: regulation of osteoblast differentiation
GO:1905114: cell surface receptor signaling pathway involved in cell-cell signaling
GO:0048812: neuron projection morphogenesis
WP447: Adipogenesis genes
ko04390: Hippo signaling pathway
GO:0030036: actin cytoskeleton organization
GO:0008285: negative regulation of cell proliferation
GO:0061061: muscle structure development
GO:0001890: placenta development
R-MMU-1266738: Developmental Biology
GO:0050769: positive regulation of neurogenesis
GO:0043588: skin development
GO:0030155: regulation of cell adhesion
-log10 (P)
D
Yap
Oct3
Gapdh
Relative expression level
E5 E3 T9
E
Yap isoform 1
1146
0
4200
1_F 1_R
2_F 2_R
3_F 3_R
4_F 4_R
Yap isoform 2
0
4152
1_F R
2_F
3_F
4_F
PCR primer
qPCR primer
F
ΔCt
E5 E3 T9
Yap isoform 1
Yap isoform 2
G
E3 T9 E5
Yap
309 bp
261 bp
Arbp

Fig. S2. Hippo pathway in PGC specification.

A) Functional enrichment analysis of the genes upregulated in E3 and T9 EpiSCs compared with E5 EpiSCs. See also Table S2.

B) Functional enrichment analysis of the genes upregulated in aggregates of E3 on day 2 and aggregates of T9 on day 2 compared with aggregates of E5 cells on day 2. See also Table S3.

C) Functional enrichment analysis of genes downregulated in aggregates of E3 on day 2 and aggregates of T9 on day 2 compared with aggregates of E5 cells on day 2. See also Table S4.

D) The expression of *Yap*, *Oct4*, and *Gapdh* in the E5, E3, and T9 cell lines as measured by RNA-Seq. Blue circle shows the average value from two independent experiments (log_2 scale).

E) Primer sets for detection of the *Yap* isoform 1 and/or 2.

F) The expression of the *Yap* isoforms 1 and 2 in the E5, E3, and T9 cell lines as measured by Q-PCR with the primer sets shown in (E).

G) RT-PCR analysis of the *Yap* isoforms 1 and 2 with the primer sets shown in (E).

Development • Supplementary information

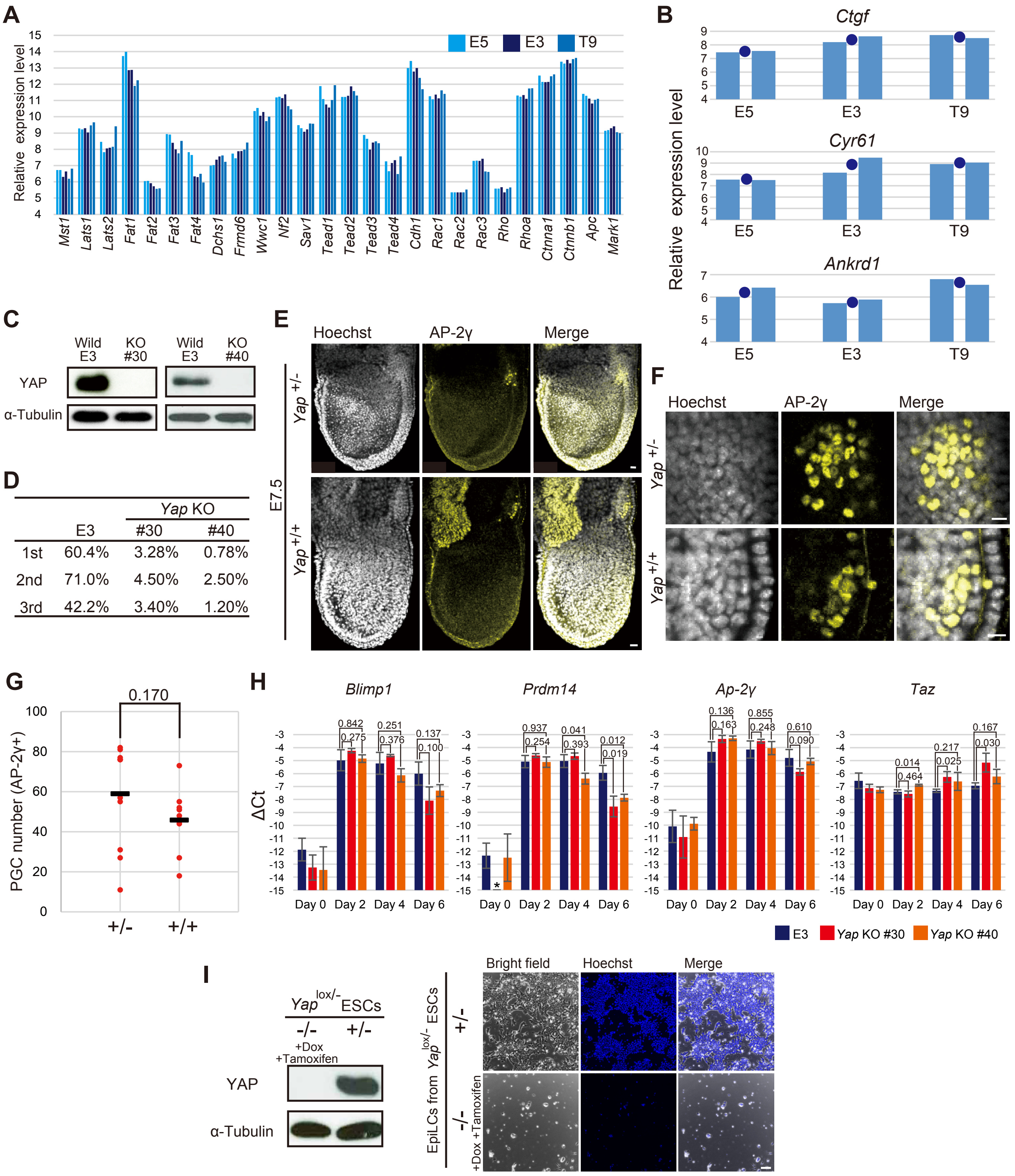

Development • Supplementary information

Fig. S3. PGC number in *Yap*$^{+/-}$ embryos do not decline.

A) The expression of the components of Hippo pathway in the E5, E3, and T9 cell lines as measured by RNA-Seq.

B) The expression of *Ctgf*, *Cyr61*, and *Ankrd1* in the E5, E3, and T9 cell lines on day 2 after cytokine stimulation for PGCLC induction as measured by RNA-Seq. Blue circle shows the average value from two independent experiments (log_2 scale).

C) Western blot analysis of YAP in *Yap*-knocked out E3 cell lines with the CRISPR-Cas9 system (*Yap* KO E3).

D) The PGCLC induction rates analyzed by FACS in Fig. 4A (three independent experiments).

E, F) Immunofluorescence analysis of AP-2γ in *Yap*$^{+/-}$ and *Yap*$^{+/+}$ embryos at E7.5. Whole epiblasts (E) and the region at the base of allantois (F) are shown. Scale bar, 10 µm.

G) The number of PGCs (AP-2γ+) in *Yap*$^{+/-}$ and *Yap*$^{+/+}$ embryos at E7.5. *P* values were calculated by two-tailed unpaired *t*-test.

H) The expression of germ cell determinant genes (*Blimp1*, *Prdm14*, and *AP-2γ*) and *Taz* in E3 and *Yap* KO E3 during PGCLC induction. *P* values were calculated by two-tailed unpaired *t*-test. *: The signal was not detected.

I) Western blot analysis (left) of YAP in *Yap*$^{+/-}$ ESCs; *Yap* can be knocked out with dox and tamoxifen through Cre-loxP recombination. Hoechst staining (right) of EpiLCs induced from *Yap*$^{+/-}$ ESCs with or without dox plus tamoxifen. Scale bar, 100 µm.

A

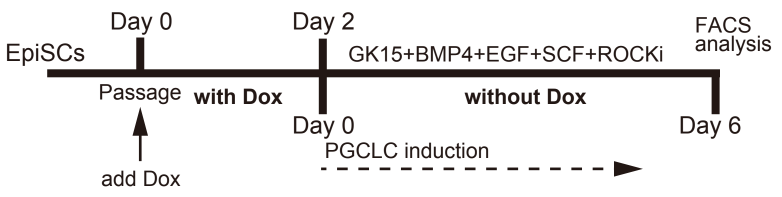

B C

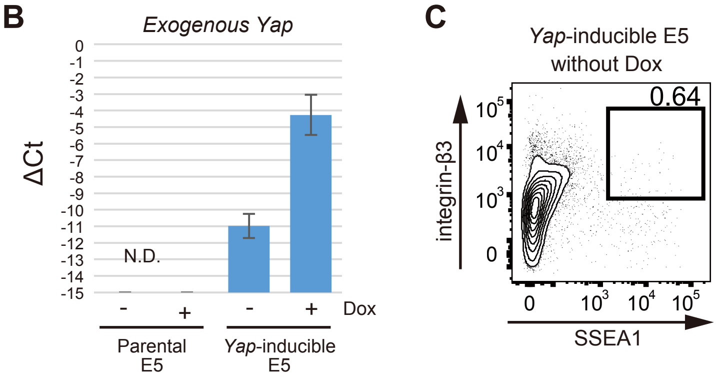

D

	Parental E5	*Yap*-inducible E5 with Dox	*Yap*-inducible E5 without Dox
1st	1.21%	21.0%	0.64%
2nd	3.20%	12.5%	1.12%

Fig. S4. Exogenous *Yap* overexpression rescues the germ cell competence in E5.

A) Scheme for PGCLC induction from *Yap*-overexpressed E5 based on the established method (Hayashi et al., 2011).

B) The expression of exogenous *Yap* in parental E5 and *Yap*-inducible E5 as measured by Q-PCR. The ΔCT from the average CT values of the two independent housekeeping genes *Arbp* and *Ppia* was calculated. The average value from three independent experiments is shown (log_2 scale).

C) Representative FACS plot images of SSEA1 and integrin β-3 expression in *Yap*-inducible E5 without dox under conditions for inducing PGCLCs from EpiSCs (two independent experiments). The percentage of the induced PGCLC population is shown. See also Figs 5A, S4D.

D) The PGCLC induction rates analyzed by FACS in Figs 5A, S1C (two independent experiments).

Table S1. Genes downregulated in E3 and T9 compared with E5 cell lines.

Click here to download Table S1

Table S2. Genes upregulated in E3 and T9 compared with E5 cell lines.

Click here to download Table S2

Table S3. Genes upregulated in D2agg_E3 and D2agg_T9 compared with D2agg_E5 cell lines.

Click here to download Table S3

Table S4. Genes downregulated in D2agg_E3 and D2agg_T9 compared with D2agg_E5 cell lines.

Click here to download Table S4

Table S5. The information of guide RNA used in this study.

Click here to download Table S5

Table S6. Primers for Q-PCR used in this study.

Click here to download Table S6
