# Supplementary material for: YAP establishes epiblast responsiveness to inductive signals for germ cell fate
Source: Development. 2021 Oct 19;148(20):dev199732. doi: 10.1242/dev.199732 (PMC8571999; doi:10.1242/dev.199732)
Supplement: Reviewer comments [file develop-148-199732_review_history.pdf]

YAP establishes epiblast responsiveness to inductive signals for germ cell fate

Saya Kagiwada, Shinya Aramaki, Guangming Wu, Borami Shin, Eva Kutejova, David Obridge, Kenjiro Adachi, Jeffrey L. Wrana, Karin Hübner and Hans R. Schöler

DOI: 10.1242/dev.199732

Editor: Maria Elena Torres-Padilla

Review timeline

Original submission:	21 April 2021
Editorial decision:	1 June 2021
First revision received:	28 July 2021
Editorial decision:	23 August 2021
Second revision received:	8 September 2021
Accepted:	9 September 2021

Original submission

First decision letter

MS ID#: DEVELOP/2021/199732

MS TITLE: YAP creates epiblast responsiveness to inductive signals for germ cell fate

AUTHORS: Saya Kagiwada, Shinya Aramaki, Guangming Wu, David Obridge, Borami Shin, Kenjiro Adachi, Eva Kutejova, Jeffrey L. Wrana, Karin Huebner, and Hans R. Schoeler

Thank you for submitting your above manuscript to Development. I have now received all the referees' reports on the above manuscript, and have reached a decision. The referees' comments are appended below, or you can access them online: please go to BenchPress and click on the 'Manuscripts with Decisions' queue in the Author Area.

As you will see, the referees express considerable interest in your work. However, they do also have some significant criticisms and recommend a substantial revision of your manuscript before we can consider publication. If you are able to revise the manuscript along the lines suggested, I will be happy receive a revised version of the manuscript.

In particular, the reviewers suggest a more thorough characterisation of the datasets, the addition of replicates to sustain robustness of some of the conclusions, as well as additional experiments. Namely, additional transcriptomic comparison between EpiSCs cultured in different conditions and formative stem cells will be required (reviewer 1) and further RNAseq analysis to analyse e.g. Hippo pathway members (reviewer 2). Significance should be evaluated throughout the results to sustain the conclusions (reviewer 2) and statistical and robust analyses for the YAP target readout (either by increasing qRT-PCR replicates and/or using the luciferase analysis suggested should be added.

Overall, both reviewer 2 and 3 raised concerns about the microscopy (e.g. YAP and histone methylation analyses), that I would encourage you to address in full.

Finally, both reviewer 1 and 2 suggest additional experiments to test the sufficiency of Yap activation in incompetent E5 EpiSCs to establish the germline competency.

© 2021. Published by The Company of Biologists under the terms of the Creative Commons Attribution License (https://creativecommons.org/licenses/by/4.0/).

The revised manuscript should address by further text amendments or clarifications, the remaining reviewers concerns including for example, providing more background information in the introduction, and a rationale for focusing on Hippo/YAP pathways (reviewers 2 and 3).

Your revised paper will be re-reviewed by one or more of the original referees, and acceptance of your manuscript will depend on your addressing satisfactorily the reviewers' major concerns. Please also note that Development will normally permit only one round of major revision.

We are aware that you may be experiencing disruption to the normal running of your lab that make experimental revisions challenging. If it would be helpful, we encourage you to contact us to discuss your revision in greater detail. If this is helpful, please send us a point-by-point response indicating where you are able to address concerns raised (either experimentally or by changes to the text) and where you will not be able to do so within the normal timeframe of a revision. We will then provide further guidance.

Considering the extent of the revisions suggested, please also note that we are happy to extend revision timeframes as necessary.

Please attend to all of the reviewers' comments and ensure that you clearly highlight all changes made in the revised manuscript. Please avoid using 'Tracked changes' in Word files as these are lost in PDF conversion. I should be grateful if you would also provide a point-by-point response detailing how you have dealt with the points raised by the reviewers in the 'Response to Reviewers' box. If you do not agree with any of their criticisms or suggestions please explain clearly why this is so.

Reviewer 1

Advance summary and potential significance to field

In this manuscript, the authors reported a novel evidence of the ability of epiblast stem cells (EpiSCs) to differentiate to primordial germ cells (PGCs). The previous reports indicated that EpiSCs at the primed pluripotent state have lost the ability to give rise PGCs (Hayashi et al, Cell, 2011). However, here the authors demonstrated that some EpiSC lines retain the ability in response to BMP4. Moreover, they found that the germline competency depends on the nuclear localization of the activated YAP protein.

The competency to differentiate into PGCs is transiently appeared during the transition of pluripotent state. The recent reports showed that the cells at the formative pluripotent state directly respond to germ cell induction (Kinoshita et al, Cell Stem Cell, 2021). The finding shown in this manuscript counteracts such dogmatic view of the transition of pluripotent state. Therefore this is important argument for the definition of each pluripotent state and the further characterization of this finding will be required in comparison to the previous observations.

Comments for the author

1. Hayashi et al reported that EpiSCs established and maintained on MEF in N2B27 containing Activin A bFGF and KSR are inefficient in the formation of PGCLCs (Cell, 2011). Here the authors applied the different culture condition: MEF-conditioned medium (CM) containing 10 ng/mL FGF2 (CMF) on dishes coated with FCS. Is the difference of the culture condition modulate the germ cell competency of EpiSCs? What happens if the germ cell competent EpiSCs (E3 and T9) are cultured in Hayshi's condition?

2. Transcriptomic comparison between EpiSCs cultured in different conditions, and the formative stem cells will be required. It will be easy using the RNA-seq data of previous publications. E3 and T9 may be closer to formative cells than primed EpiSCs.

3. The authors showed the necessity of Yap on the germ cell induction in germ cell competent E3 EpiSCs.
How about the sufficiency of Yap activation in incompetent E5 EpiSCs to establish the germline competency?

© 2021. Published by The Company of Biologists under the terms of the Creative Commons Attribution License (https://creativecommons.org/licenses/by/4.0/).

4. In Fig 3C, the cytoplasmic localization of YAP in E5 EpiSCs is equivocal. The positions indicated by arrowheads lack both YAP and Hoechst signals. Clear microscopic data as well as the quantification of nuclear localization signals (% of nuclei) should be shown.

Reviewer 2

Advance summary and potential significance to field

In this manuscript Kagiwada et al. examine whether different epiblast stem cell lines are competent for primordial germ cell like cell fate specification and characterize the molecular underpinnings of the observed differences. While it is an intriguing finding that certain EpiSC lines indeed give rise to PGCLCs, the involvement of Yap in this process is much less clear. Additionally, a number of controls are missing in experiments which make it difficult to interpret their significance.

Comments for the author

Major points:
Figure 2: epigenetic characterization – please include positive controls for immunofluorescent stainings of PGCLCs differentiated from EpiLCs.
The induction of primary oocytes also needs negative (ESC, non-GC-competent EpiSC line) and positive controls (EpiLC-derived PGCLCs).

Inconsistency in the percent of PGCLCs induced in Figure 1: Figure 1 A and C have panels that show the same experimental conditions (PGCLC induction from EpiSCs using all factors analyzed by FACS on day 6) – yet the percentages of induced PGCLCs are very different. Why is this? There is also inconsistency in some of these numbers and what is written in the text. For example, PGCLC induction from EpiLCs is 15% in the text but I do not see this in the figure.

The argument for selecting the study of the Hippo pathway based on the downregulation of genes involved in “tissue morphology”, “cytoskeleton”, and “the response to stress” is very unconvincing. That a difference in Yap localization exists between germ line competent and non-copetent EpiSCs is not convincing. Immunofluorescent stainings should be quantified for N/C Yap ratios, stainings should be done at least in three independent replicates, as they can be highly variable. Moreover phospho-Yap, a better readout for Hippo activity, should also be visualized and quantified. Western blot for phospho-Yap between competent and non-competent cell lines would also help confirm differential Hippo activity.

Is Yap also required in EpiLCs for germline inducing PGCLCs, or is this a specific feature distinguishing only different EpiSC lines?
There is a discrepancy between the PGCLC and PGC forming ability of Yap mutant EpiSCs and embryos, respectively. Could Taz be compensating in the embryo, but not in EPISCs? Could the authors look at Taz expression?

Please show RNA-sequencing in Figure 3A as a PCA plot as well. Overall day 2 stimulated aggregates of all EpiSC lines (both GC competent and not competent)
cluster closer together than day 2 stimulated EpiLCs. I do not see how this data supports that germ line copetent and non-competent EPICSs are different in their response to cytokines.
Please show RNA-sequencing in Figure 4E as a PCA plot as well. The text states that cytokine simulation clusters cell lines together based on their competence but in this figure I only see unstimulated cells. Was PGCLC induction performed on Yap KO cells and analyzed by RNA seq?

Perhaps my biggest concern is that Yap was shown to be required for differentiation of ESCs, including into mesoderm (Chung et al 2016; Sun et al. 2020). Therefore, could the observed defects in Yap mutant EPISCs be due to a general differentiation problem, rather than a defect specifically associated with PGC formation? Data shown in Figure 4F support this notion. The focus of the manuscript, which is now on PGC formation, should be re-evaluated.
Chung et al. Yap1 is dispensable for self-renewal but required for proper differentiation of mouse embryonic stem (ES) cells. EMBO Rep. 2016 Apr;17(4):519-29.

© 2021. Published by The Company of Biologists under the terms of the Creative Commons Attribution License (https://creativecommons.org/licenses/by/4.0/).

Sun et al. Hippo-YAP signaling controls lineage differentiation of mouse embryonic stem cells through modulating the formation of super-enhancers, Nucleic Acids Research, Volume 48, Issue 13, 27 July 2020, Pages 7182–7196

Reviewer 3

Advance summary and potential significance to field

This manuscript detailed the differences in germline competency between 3 EpiSC lines. The authors identified that 2 of the 3 lines (E3, T9) show competency, while E5 is unable to generate PGCLC in culture. Through differential RNA-Seq analysis the authors propose that the Hippo pathway, and specifically YAP1 may be regulating germline competency. YAP1 knockout experiments show reduced competency in the E3 line, while Yap1 null mice show reduced PGC populations in vivo. The authors hypothesis that nuclear expression of YAP1 allows for PGCLC pathways to be upregulated, while cytoplasmic expression reduces expression of pathway genes, leading to no or minimal germline competency.
This work will be of interest to stem cell researchers, particularly those that work on germline competency and the generation of PGCLCs.

Comments for the author

While it has been shown that different EpiSC lines show different levels of germline competency, further experimentation may be required to show the role of Yap1 during this developmental process.

Major Points
1. One of the biggest issues with the current manuscript is the overall preliminary feeling generated by the datasets. A large part of this comes from the almost complete lack of statistical analysis. For all quantitative analyses there are no statistical tests performed as seen in Fig 3D, Fig 4D Fig 4F, Fig 4D, Fig EV3 4D. The authors should perform statistical analyses on this data, as it strengthen the conclusions if significantly different.

2. YAP downregulation was shown to decrease the germline competency however to fully investigate the role of YAP – useful and informative experiments would include rescue experiments where YAP is expressed in the nucleus of the E5 line and germline competency assessed. This could be achieved through generation of an ERT2 line which can specifically direct expression of a target gene to the nucleus.

3. To understand the role of YAP during germ cell development, direct experiments to assess YAP activity within these cells should be performed to strengthen the study. While the authors have performed analyses of YAP target genes CTGF and CYR61 no statistical tests (such as t-test) were performed.
Repeating this with more targets through qRT-PCR with a $n \geq 3$, allowing for robust analyses would be beneficial. A more direct method of assessing the involvement of YAP/TAZ activity during germ cell differentiation would be to use established luciferase assays. These can contain; CTGF, ANKRD1, or RHAMM/HMMR promoter regions, multimerised TEAD binding elements or multimerised GAL4 binding elements. The book chapter 'Luciferase Reporter Assays to Determine YAP/TAZ Activity in Mammalian Cells' (Dupont, 2018 - PMID: 30565131) contains detailed information on such experiments.

4. There is no mention of the Yap-/- mouse line in the materials and methods. Was this generated in house or from a previous study? Further, was this bred with the Blimp1-RFP mouse line? The authors should include these details in the text. If this line was generated for this study, a diagram of the line generation should be included, as well as confirmation of Yap1 knockout/deletion through qPCR/Western Blot or sequence analysis.

5. To understand why there are differences between the 3 EpiSC lines RNA-Seq or qPCR data of components of the Hippo pathway will be informative.

© 2021. Published by The Company of Biologists under the terms of the Creative Commons Attribution License (https://creativecommons.org/licenses/by/4.0/).

If there are differences within the pathway components this could show why YAP is expressed differently between the lines.

6. Yap1 has different isoforms. Can the authors confirm if each of the lines expresses the same isoform as this may be one of the reasons for differential expression between the lines.

7. The analysis of 5mc, H3k9me2 and H3k27me3 through a single IF stain is not sufficient to draw strong conclusions. Further experiments such as ChIP-Seq and Western blotting are required.

8. Fig 3C – there still appears to be nuclear staining of Yap in the E5 line, as well as cytoplasmic expression with E3 and T9. As the pathway proposed by the authors centres around YAP expression either within the nucleus or cytoplasm this is an important distinction. As only 1 sample image is shown, the authors should repeat the experiment with more samples in order to quantify if the levels of YAP differ significantly between the lines. It has previously been noted in the literature that fibronectin interferes with YAP signalling and should be avoided in IF based assays for coating glass. The methods section does not indicate if the authors used fibronectin to coat the glass bottom dishes. If fibronectin was used the authors should repeat this experiment using either Poly-D-Lysine or laminin to coat the glass to ensure no confounding effects are seen from the fibronectin(Rausch & Hansen 2019 'Immunofluorescence study of endogenous YAP in mammalian cells' PMID: 30565128) Further to this, there appears to be a difference in nuclei size and cell density between the E5 and E3 and T9 lines. Such differences could play into the YAP pathway, so the authors should mention this in the text and openly discuss any phenotypic differences observed in these lines.

9. The PGC analyses in the Yap mouse line does not provide enough evidence to use the Yap +/- line as the control for Fig 4B and 4D. In the comparison between Yap +/+ and Yap +/- embryos (EV3B), of the representative images shown these embryos appear to be at different stages of development as seen by the location of the AP-2γ stained cells. If the quantification of the germ cell number has been generated from different stages this may not be an accurate representation between the genotypes. The authors state that there is no obvious effects on PGC numbers in Yap +/- mice, however this quantification of this data (Fig EV3 D) has not been tested for significance. This data should be analysed via a t-test. As the figure currently is, the mean of the heterozygous animals is higher than the wildtype. If the authors perform these tests and show a difference, they will need to hypothesise why heterozygous animals would have increased PGC numbers as this is not in line with their other observations and conclusions.
The outcome of these tests will contribute to the further analysis shown in Figure 3D. If there are any significant differences found between Yap +/+ and Yap +/- PGC numbers, the wildtype Yap +/+ should be included in the quantification of Blimp1-RFP, AP-2γ PGCs. This data also needs to be analysed by a t-test.

10. To determine differences between the lines, the E5 EpiSCs should be included in the qPCR analysis in Fig 1B. This will help to identify if there are any differentially expressed genes in the EpiSCs and after differentiation. The authors note there are no differences in expression of genes between the E3 and T9 lines when assessed via qPCR, however the flow cytometry data from PGCLC differentiation appears quite different with much higher levels of SSEA1 expression seen in the T9 line compared to E3. Can the authors include this in the qPCR gene panel and determine if these differences are present within the EpiSC population.

11. The references within the introduction relating to the involvement of WNT/ß-Catenin signalling in germ cell development are overstated. Line 49-54 states;
'In mice, a co-operative regulatory mechanism between the BMP/SMAD and WNT/ß-CATENIN signalling pathways has been studied extensively and demonstrated that these two pathways are critical for the induction of germ cells and other neighbouring somatic lineages in nascent mesoderm derived from pluripotent epiblast'
The evidence for BMP signalling involvement in germ cell development is much stronger than that for WNT/ß-CATENIN and this should be reflected in the language used, as the authors propose these have a similar impact. Further the references for WNT signalling involvement (Liu 1999, Huelskin 2000 and Winnier 1996) should not be cited here as they do not include germ cell phenotypes. The authors should rework this section of the introduction to more accurately reflect the literature on WNT and BMP involvement in germ cell development.

© 2021. Published by The Company of Biologists under the terms of the Creative Commons Attribution License (https://creativecommons.org/licenses/by/4.0/).

1. Table S1 – To facilitate re-use of the data, this should be provided as an excel spreadsheet rather than PDF download

Minor Points

2. Although the paper conclusions centre around the role of YAP in germline competency, this is not mentioned within the introduction. Inclusion of some background information within the introduction would be beneficial to the reader for contextualisation of the experiments.

3. There have been some previous reports of YAP involvement in the germline. It would be useful to have these in the discussion to support the arguments for YAP involvement in this context. For example, in Ye (2017, PMID: 28245464) Yap1 was overexpressed and downregulated in ovarian germline stem cells (OGSCs). Germ cell markers were upregulated in Yap1 overexpressing OGSC and mice showed follicular regeneration and increased birth rate. While shRNA knockdown led to reduced proliferation in cells, decreased follicules in mouse ovaries, and reduced birth rates indicating Yap involvement in ovarian germ cell development.

4. What are the main differences between the 3 EpiSC lines? Are they all from the same mouse background – this should be included in the text.

5. In the PGCLC differentiation experiments there is no indication of how many times these have been performed or if the results are from a single experiment.

6. Fig 1B -It is difficult to determine if these cells have been sorted prior to qPCR analysis.

7. Fig 1D – there is no EpiLC control for these lines.

8. Fig 3A, Fig 4E- These figures would be much more readable if turned 90 degrees.

9. Fig 3A – the PGCLCs from E3 and T9 are more similar to in vivo PGCs than ESC derived PGCLCs than would be expected. Can the authors comment on why they think this is the case.

10. Figure 4H – figure has a spelling error, should be 'nucleus'

11. Fig EV1 – The diagram includes GFP being added to the PGC differentiation media – this may be a typo? Can the authors also explain why ROCK inhibitor was used as this is not standard practice for mouse PGCLC differentiation.

12. Line 118 – the specific numbers of PGCLCs is given. This should be presented as a percentage of total cells as this is variable between samples.

13. Line 177 – the authors should expand on what the component of the pathway they are referring to is.

14. Line 224 – The authors should clarify that they have shown YAP contributes to germ cell specification through WNT signalling in this specific context, rather than in general.

15. Line 231 – Previous studies are alluded to but references are missing.

16. Line 297 – details should be published in this paper if it is published first, otherwise link to preprint.

17. Line 379 - Should read RNA Sequencing

© 2021. Published by The Company of Biologists under the terms of the Creative Commons Attribution License (https://creativecommons.org/licenses/by/4.0/).

First revision

Author response to reviewers' comments

Rebuttal: DEVELOP/2021/199732

We would like to sincerely thank the referees for their encouraging and constructive comments, which have been instrumental in helping us produce our revised version of the manuscript.

Reviewer comments:
Reviewer #1:
Reviewer 1 Advance summary and potential significance to field
In this manuscript, the authors reported a novel evidence of the ability of epiblast stem cells (EpiSCs) to differentiate to primordial germ cells (PGCs). The previous reports indicated that EpiSCs at the primed pluripotent state have lost the ability to give rise PGCs (Hayashi et al, Cell, 2011). However, here the authors demonstrated that some EpiSC lines retain the ability in response to BMP4. Moreover, they found that the germline competency depends on the nuclear localization of the activated YAP protein. The competency to differentiate into PGCs is transiently appeared during the transition of pluripotent state. The recent reports showed that the cells at the formative pluripotent state directly respond to germ cell induction (Kinoshita et al, Cell Stem Cell, 2021). The finding shown in this manuscript counteracts such dogmatic view of the transition of pluripotent state. Therefore this is important argument for the definition of each pluripotent state and the further characterization of this finding will be required in comparison to the previous observations.

Response 1. We would like to sincerely thank the Reviewer for his/her encouraging comments on our manuscript. In the revised edition, we have addressed the suggested issues to improve our manuscript.

Reviewer 1 Comments for the author

1.Hayashi et al reported that EpiSCs established and maintained on MEF in N2B27 containing Activin A, bFGF and KSR are inefficient in the formation of PGCLCs (Cell, 2011). Here the authors applied the different culture condition: MEF-conditioned medium (CM) containing 10 ng/mL FGF2 (CMF) on dishes coated with FCS. Is the difference of the culture condition modulate the germ cell competency of EpiSCs? What happens if the germ cell competent EpiSCs (E3 and T9) are cultured in Hayshi’s condition?

Response 2. Following to the Reviewer’s suggestion, we cultured E3 and T9 under Hayashi’s condition (N2B27 medium with 20% KSR, 20 ng/mL of activin A, and 12 ng/mL of bFGF) to examine the condition’s effect on PGCLC induction from these EpiSC lines. As shown in Fig. S1C, E3 and T9 have shown much less germ cell competence under this condition, suggesting that undefined factors in CM are critical for maintaining competence. We have incorporated a relevant passage into the revised manuscript (Lines 120-125 of text).

2.Transcriptomic comparison between EpiSCs cultured in different conditions, and the formative stem cells will be required. It will be easy using the RNA-seq data of previous publications. E3 and T9 may be closer to formative cells than primed EpiSCs.

Response 3. In accordance with the Reviewer’s suggestion, we compared the transcriptomes of EpiSC lines cultured under different conditions (i.e., this study and Hayashi’s paper). The PCA analysis revealed that incompetent EpiSCs cultured under Hayashi’s condition show a relatively different transcriptional profile compared with other competent EpiSC lines or EpiLCs. We have incorporated the data and a relevant passage into the revised manuscript (Fig. S1E, Lines 120-127 of text).

3.The authors showed the necessity of Yap on the germ cell induction in germ cell competent E3 EpiSCs. How about the sufficiency of Yap activation in incompetent E5 EpiSCs to establish the germline competency?

© 2021. Published by The Company of Biologists under the terms of the Creative Commons Attribution License (https://creativecommons.org/licenses/by/4.0/).

Response 4. In the revised manuscript, we have examined the effect of Yap overexpression in non-GC-E5 EpiSCs on competence and found that exogenous Yap clearly restores the competence of E5 EpiSCs (Fig. 5A, Lines 274-286 of text).

4.In Fig 3C, the cytoplasmic localization of YAP in E5 EpiSCs is equivocal. The positions indicated by arrowheads lack both YAP and Hoechst signals. Clear microscopic data as well as the quantification of nuclear localization signals (% of nuclei) should be shown.
Response 5. According to the Reviewer's suggestion, we have modified the presented microscopic image by circling the nuclei with dotted lines to clearly delineate the localization of YAP (Fig. 3D). Furthermore, we quantified the N/C (nuclear/cytoplasmic) YAP ratios in the examined EpiSC lines to evaluate the localization of YAP. This clearly revealed that there is higher nuclear localization of YAP in competent EpiSCs compared with the incompetent lines (Fig. 3E).

Reviewer 2 Advance summary and potential significance to field
In this manuscript Kagiwada et al. examine whether different epiblast stem cell lines are competent for primordial germ cell like cell fate specification and characterize the molecular underpinnings of the observed differences. While it is an intriguing finding that certain EpiSC lines indeed give rise to PGCLCs, the involvement of Yap in this process is much less clear. Additionally, a number of controls are missing in experiments which make it difficult to interpret their significance.

Response 1. We would like to sincerely thank the Reviewer for his/her constructive comments to improve our manuscript.

Reviewer 2 Comments for the author

Major points:

Figure 2: epigenetic characterization – please include positive controls for immunofluorescent stainings of PGCLCs differentiated from EpiLCs. The induction of primary oocytes also needs negative (ESC, non-GC-competent EpiSC line) and positive controls (EpiLC-derived PGCLCs).

Response 2. In accordance with the Reviewer's suggestion, we have included the 5mC, H3K9me2, and H3K27me3 staining data of PGCLCs derived from EpiLCs as a positive control (Fig. 2B). For the induction of primary oocytes, we have included data on ESCs (negative control) and EpiLC-derived PGCLCs (positive control) (Fig. 2G). As the PGCLC induction rate from E5 EpiSCs is quite low (Fig. 1A), precluding feasible analysis of further oocyte induction, we have not included the data of oocyte induction from incompetent E5 EpiSCs.

Inconsistency in the percent of PGCLCs induced in Figure 1: Figure 1 A and C have panels that show the same experimental conditions (PGCLC induction from EpiSCs using all factors analyzed by FACS on day 6) – yet the percentages of induced PGCLCs are very different. Why is this? There is also inconsistency in some of these numbers and what is written in the text. For example, PGCLC induction from EpiLCs is 15% in the text but I do not see this in the figure.

Response 3. We would like to emphasize that the PGCLC induction rate can vary across distinct lots of MEFs and their derived MEF-conditioned medium with undefined factors. We think that this might contribute or even cause the observed variability in the induction or growth rate of PGCLCs as well as other uncharacterized cells. This sometimes may contribute to the poor induction rate even in GC-EpiSCs (E3 and T9). For this reason, we consistently used the CM lot that induced more than 20% of PGCLCs. We presented the different induction rates for the distinct lots of CM (Fig. S1J) and discussed this issue in the revised manuscript (Lines 305-308 of text).
Regarding the inconsistent description of the induction rate of EpiLC-derived PGCLCs, we have deleted the induction efficiency (%) in the text and presented the rate in each figure (Fig. S1B, D, and F-J, Fig. S3D, Fig. S4D) to show the induction data in each individual replicate.

The argument for selecting the study of the Hippo pathway based on the downregulation of genes involved in "tissue morphology", "cytoskeleton", and "the response to stress" is very unconvincing. That a difference in Yap localization exists between germ line competent and non-copetent EpiSCs is not convincing. Immunofluorescent stainings should be quantified for N/C Yap ratios, stainings

© 2021. Published by The Company of Biologists under the terms of the Creative Commons Attribution License (https://creativecommons.org/licenses/by/4.0/).

should be done at least in three independent replicates, as they can be highly variable. Moreover phospho-Yap, a better readout for Hippo activity, should also be visualized and quantified.Western blot for phospho-Yap between competent and non-competent cell lines would also help confirm differential Hippo activity.

Response 4. In accordance with the Reviewer's suggestion, we quantified the N/C (nuclear/cytoplasmic) YAP ratios in the examined GC- or non-GC-EpiSC lines in the revised manuscript. This analysis revealed a higher nuclear localization of YAP in competent EpiSCs compared with the incompetent lines (Fig. 3E). Furthermore, we have also seen phospho-YAP protein together with pan-YAP in western blot analysis. We found that both forms of YAP protein are clearly activated in GC-EpiSC lines (E3 and T9) but noticeably affected in non-GC E5 EpiSCs, suggesting that there is more active Hippo signaling and its driven degradation of YAP in non-GC EpiSCs. We have incorporated these data and a relevant passage into the revised manuscript (Fig. 3G, Lines 220-235 of text).

Is Yap also required in EpiLCs for germline inducing PGCLCs, or is this a specific feature distinguishing only different EpiSC lines

Response 5. We have attempted to examine the function of Yap in EpiLC/PGCLC-differentiation context. However, inducible deletion of Yap led to massive EpiLC death, precluding further analysis. Although the exact reason for this context-specific phenomenon is not clear, we have described this observation in the revised manuscript (Fig. S3I, Lines 241-244 of text).

There is a discrepancy between the PGCLC and PGC forming ability of Yap mutant EpiSCs and embryos, respectively. Could Taz be compensating in the embryo, but not in EPISCs? Could the authors look at Taz expression?

Response 6. We consider that it is unclear whether there is a discrepancy between the forming ability of Yap-mutant PGCLCs (in vitro) and PGCs (in vivo) based on our findings here, as it is difficult to evaluate the equivalence of the developmental stage of Yap-mutant PGCs and PGCLCs. Our data actually shows that poor induction of PGCLCs is still observed in Yap-mutant EpiSCs (Fig. 4A) as well as in vivo PGCs in Yap-mutant embryos (Fig. 4B-D). Furthermore, additional Q-PCR analysis also indicates that expression of Taz and early germ cell determinants such as Blimp1 is not affected in Yap-mutant EpiSCs at early time points (day 0, 2, and 4) after cytokine stimulation (Fig. S3H), suggesting that Taz or other factors may compensate for the competence in Yap-mutant EpiSCs. We have included a corresponding passage in the revised manuscript (Lines 327-332 of text).

Please show RNA-sequencing in Figure 3A as a PCA plot as well. Overall day 2 stimulated aggregates of all EpiSC lines (both GC competent and not competent) cluster closer together than day 2 stimulated EpiLCs. I do not see how this data supports that germ line copetent and non-competent EPICSs are different in their response to cytokines.

Response 7. According to the Reviewer's suggestion, we have also presented the PCA plot in the revised manuscript (Fig. 3A). Regarding the transcriptomic profile of the response to cytokines, our statement was incorrect, as the Reviewer pointed out, and thus we have deleted the relevant text from the manuscript.

Please show RNA-sequencing in Figure 4E as a PCA plot as well. The text states that cytokine simulation clusters cell lines together based on their competence, but in this figure I only see unstimulated cells. Was PGCLC induction performed on Yap KO cells and analyzed by RNA seq?

Response 8. We have also presented the PCA plot in the revised manuscript (Fig. 4E). Regarding the statement about the cluster of stimulated aggregates based on competence, our description was incorrect, as the Reviewer pointed out, and thus we have deleted the relevant passage from the manuscript.

Perhaps my biggest concern is that Yap was shown to be required for differentiation of ESCs, including into mesoderm (Chung et al 2016; Sun et al. 2020). Therefore, could the observed defects in Yap mutant EPISCs be due to a general differentiation problem, rather than a defect specifically

© 2021. Published by The Company of Biologists under the terms of the Creative Commons Attribution License (https://creativecommons.org/licenses/by/4.0/).

associated with PGC formation? Data shown in Figure 4F support this notion. The focus of the manuscript, which is now on PGC formation, should be re-evaluated.

Chung et al. Yap1 is dispensable for self-renewal but required for proper differentiation of mouse embryonic stem (ES) cells. EMBO Rep. 2016 Apr;17(4):519-29.

Sun et al. Hippo-YAP signaling controls lineage differentiation of mouse embryonic stem cells through modulating the formation of super-enhancers, Nucleic Acids Research, Volume 48, Issue 13, 27 July 2020, Pages 7182–7196

Response 9. Although many previous studies have suggested that mesodermal program orchestrated by mesodermal TFs is an unnecessary byproduct associated with gastrulation due to its eventual inactivation (Kurimoto et al., 2008; Ohinata et al., 2005), some recent studies have clearly shown that pan-mesodermal transcription factors activated by upstream WNT signaling are required for the onset of germ cell fate induction (Aramaki et al., 2013; Chen et al., 2017; Kojima et al., 2017). Indeed, the classical WNT target T/Brachyury is required for PGC specification and directly regulates germ cell determinants (Aramaki et al., 2013). Another recent study has also shown that specific levels of T and its related residual pluripotency factors co-regulate specific enhancer loci of determinants (Blimp1 and Prdm14) to segregate the germline from somatic lineages (Aramaki et al., 2021). These findings strongly suggest that germ cell specification is not clearly separated from general or pan-mesoderm development, including somatic mesodermal tissues, but rather it is induced by common mesodermal TFs at the onset of gastrulation, which is contrary to the previous notion. Thus, based on our data (Fig. 4G, H, and Fig. 5B) and previous findings above, we consider it logical to assume that Yap creates epiblast responsiveness by mediating mesodermal WNT signaling for PGC specification as well as for other somatic mesoderm lineages. To show this notion, we have modified the description in the revised manuscript (Lines 53-67, 336-349 of text). Furthermore, we also speculate that more fine-tuned regulatory mechanisms, possibly mediated through other factors, are required for the segregation of specific germline cells from somatic mesodermal cells also induced by the YAP/WNT pathway—and thus we have discussed this in the revised manuscript (Lines 345-349 of text).

Reviewer 3 Advance summary and potential significance to field
This manuscript detailed the differences in germline competency between 3 EpiSC lines. The authors identified that 2 of the 3 lines (E3, T9) show competency, while E5 is unable to generate PGCLC in culture. Through differential RNA-Seq analysis the authors propose that the Hippo pathway, and specifically YAP1 may be regulating germline competency. YAP1 knockout experiments show reduced competency in the E3 line, while Yap1 null mice show reduced PGC populations in vivo. The authors hypothesis that nuclear expression of YAP1 allows for PGCLC pathways to be upregulated, while cytoplasmic expression reduces expression of pathway genes, leading to no or minimal germline competency. This work will be of interest to stem cell researchers, particularly those that work on germline competency and the generation of PGCLCs.

Reviewer 3 Comments for the author
While it has been shown that different EpiSC lines show different levels of germline competency, further experimentation may be required to show the role of Yap1 during this developmental process.

Response 1. We would like to sincerely thank the Reviewer for his/her constructive comments on our manuscript.

Major Points
1.One of the biggest issues with the current manuscript is the overall preliminary feeling generated by the datasets. A large part of this comes from the almost complete lack of statistical analysis. For all quantitative analyses there are no statistical tests performed as seen in Fig 3D, Fig 4D, Fig 4F, Fig 4D, Fig EV3 4D. The authors should perform statistical analyses on this data, as it strengthen the conclusions if significantly different.

Response 2. In accordance with the Reviewer’s suggestion, we performed quantification with statistical analysis in the indicated data of the manuscript (Fig. 3H, Fig. 4D, Fig. 4G, Fig. S3G in the revised manuscript) (Fig. 3D, Fig. 4D, Fig. 4F, Fig. EV3 4D in the original manuscript).

© 2021. Published by The Company of Biologists under the terms of the Creative Commons Attribution License (https://creativecommons.org/licenses/by/4.0/).

2.YAP downregulation was shown to decrease the germline competency, however to fully investigate the role of YAP – useful and informative experiments would include rescue experiments where YAP is expressed in the nucleus of the E5 line and germline competency assessed. This could be achieved through generation of an ERT2 line which can specifically direct expression of a target gene to the nucleus.

Response 3. According to the Reviewer's suggestion, we examined the rescue experiment by using the Tet-on inducible system and found that Yap overexpression in incompetent E5 EpiSCs restores competence. We have incorporated these data and a corresponding passage into the revised manuscript (Fig. 5A,B, and Fig. S4B,C, Lines 274-286 of text).

3.To understand the role of YAP during germ cell development, direct experiments to assess YAP activity within these cells should be performed to strengthen the study. While the authors have performed analyses of YAP target genes CTGF and CYR61 no statistical tests (such as t-test) were performed. Repeating this with more targets through qRT-PCR with a $n \geq 3$, allowing for robust analyses would be beneficial. A more direct method of assessing the involvement of YAP/TAZ activity during germ cell differentiation would be to use established luciferase assays. These can contain; CTGF, ANKRD1, or RHAMM/HMMR promoter regions, multimerised TEAD binding elements or multimerised GAL4 binding elements. The book chapter 'Luciferase Reporter Assays to Determine YAP/TAZ Activity in Mammalian Cells' (Dupont, 2018 - PMID: 30565131) contains detailed information on such experiments.

Response 4. In accordance with the Reviewer's suggestion, we performed Q-PCR analysis of 3 target genes with n=3 in the revised manuscript (Fig. 3H). Furthermore, we examined transcriptional activity by using the luciferase reporter regulated under the control of repetitive TEAD responsive elements and the promoter sequence of CTGF. These analyses clearly revealed that YAP activity is more enhanced in GC-EpiSCs (E3 and T9) compared with non-GC-EpiSCs (E5) (Fig. 3I). We have also incorporated a corresponding passage into the revised manuscript (Lines 227-235 of text).

4.There is no mention of the Yap-/- mouse line in the materials and methods. Was this generated in house or from a previous study? Further, was this bred with the Blimp1-RFP mouse line? The authors should include these details in the text. If this line was generated for this study, a diagram of the line generation should be included, as well as confirmation of Yap1 knockout/deletion through qPCR/Western Blot or sequence analysis.

Response 5. We thank the Referee for pointing this out. We used previously established Yap-mutant mice (Reginensi et al., 2013) bred with the Blimp1-RFP line (Sugimoto and Abe, 2007). We have incorporated these references into the revised manuscript (Lines 369-376 of text).

5.To understand why there are differences between the 3 EpiSC lines, RNA-Seq or qPCR data of components of the Hippo pathway will be informative. If there are differences within the pathway components this could show why YAP is expressed differently between the lines.

Response 6. According to the Reviewer's suggestion, we have presented RNA-Seq data of major Hippo signaling components in the revised manuscript. However, there are no obvious differences in such factors between these three EpiSC lines (Fig. S3A), suggesting that other factors or regulatory mechanisms are involved in the distinct localization of YAP in EpiSC lines. We have included a relevant passage into the revised manuscript (Lines 217-219 of text). Related to this, as we observed that GC- and non-GC-EpiSC lines exhibit nuclei of different size, in accordance with the Reviewer's suggestion, we have discussed the possibility that this might cause distinct Hippo activation (Fig. 3F, Lines 222-224 of text). Please also see Response 9 described below for further details.

6.Yap1 has different isoforms. Can the authors confirm if each of the lines expresses the same isoform as this may be one of the reasons for differential expression between the lines.

Response 7. In response to the Reviewer's suggestion, we performed Q-PCR analysis to examine the two different spliced isoforms previously identified. However, the analysis revealed that these two

© 2021. Published by The Company of Biologists under the terms of the Creative Commons Attribution License (https://creativecommons.org/licenses/by/4.0/).

isoforms show similar expression level. We have incorporated the data and a corresponding passage into the revised manuscript (Fig. S2E-G, Lines 213-216 of text).

7.The analysis of 5mc, H3k9me2 and H3k27me3 through a single IF stain is not sufficient to draw strong conclusions. Further experiments such as ChIP-Seq and Western blotting are required.

Response 8. In accordance with the Reviewer's suggestion, we performed western blot analysis of H3K9me2 and H3K27me3. To further confirm the result, we also quantified the band intensities. These analyses clearly revealed the downregulation of H3K9me2 and the upregulation of H3K27me3 in PGCLCs (Fig. 2D-F). Regarding 5mC, as collection of a sufficient number of PGCLCs for western blot analysis of 5mC is not feasible, we have only shown the quantification data with statistical analysis of immunofluorescence intensity, which also revealed that 5mC is significantly repressed in the PGCLC population (Fig. 2B,C).

8.Fig 3C – there still appears to be nuclear staining of Yap in the E5 line, as well as cytoplasmic expression with E3 and T9. As the pathway proposed by the authors centres around YAP expression either within the nucleus or cytoplasm this is an important distinction. As only 1 sample image is shown, the authors should repeat the experiment with more samples in order to quantify if the levels of YAP differ significantly between the lines. It has previously been noted in the literature that fibronectin interferes with YAP signalling and should be avoided in IF based assays for coating glass. The methods section does not indicate if the authors used fibronectin to coat the glass bottom dishes. If fibronectin was used the authors should repeat this experiment using either Poly-D-Lysine or laminin to coat the glass to ensure no confounding effects are seen from the fibronectin(Rausch & Hansen 2019 'Immunofluorescence study of endogenous YAP in mammalian cells' PMID: 30565128) Further to this, there appears to be a difference in nuclei size and cell density between the E5 and E3 and T9 lines. Such differences could play into the YAP pathway, so the authors should mention this in the text and openly discuss any phenotypic differences observed in these lines.

Response 9. According to the Reviewer's suggestion, we quantified the N/C (nuclear/cytoplasmic) YAP ratios in the examined EpiSC lines to evaluate the localization of YAP (three independent images in each EpiSC line). This analysis revealed the higher nuclear localization of YAP in competent EpiSCs compared with incompetent lines (Fig. 3E). In addition, we measured the nuclei size of these EpiSC lines and found that GC-EpiSCs exhibited nuclei of smaller size compared with non-GC-EpiSCs, suggesting that a regulatory mechanism may account for the different Hippo signal activity, leading to distinct YAP localization. We have included the data and a corresponding passage into the revised manuscript (Fig. 3F, Lines 223-225 of text). Regarding the coating method, we used FCS, as described in the Materials and Methods (Line 473 of text), but not fibronectin in all the culture experiments of EpiSCs in this study.

9.The PGC analyses in the Yap mouse line does not provide enough evidence to use the Yap +/- line as the control for Fig 4B and 4D. In the comparison between Yap +/+ and Yap +/- embryos (EV3B), of the representative images shown these embryos appear to be at different stages of development as seen by the location of the AP-2γ stained cells. If the quantification of the germ cell number has been generated from different stages this may not be an accurate representation between the genotypes. The authors state that there is no obvious effects on PGC numbers in Yap +/- mice, however this quantification of this data (Fig EV3 D) has not been tested for significance. This data should be analysed via a t-test. As the figure currently is, the mean of the heterozygous animals is higher than the wildtype. If the authors perform these tests and show a difference, they will need to hypothesise why heterozygous animals would have increased PGC numbers as this is not in line with their other observations and conclusions. The outcome of these tests will contribute to the further analysis shown in Figure 3D. If there are any significant differences found between Yap +/+ and Yap +/- PGC numbers, the wildtype Yap +/+ should be included in the quantification of Blimp1-RFP, AP-2γ PGCs. This data also needs to be analysed by a t-test.

Response 10. To ensure that we analyze embryos of the same developmental stage, we collected embryos from the same litter, which is a standard method in the relevant field. We speculate that the different appearance of the embryos analyzed has to do with the abnormal or incomplete mesodermal development in Yap-mutant embryos, which has been reported in the original paper of Yap-mutant embryos (Morin-Kensicki et al., 2006). Actually, in Yap-mutant embryos,

© 2021. Published by The Company of Biologists under the terms of the Creative Commons Attribution License (https://creativecommons.org/licenses/by/4.0/).

extraembryonic mesodermal tissues, such as allantois and yolk sac neighboring the initial specified PGCs, are also affected. Importantly, recent studies have shown that some mesodermal specifiers, such as T/Brachyury, directly regulate germ cell determinants and are required for induction of the germ cell lineage as well as other mesodermal lineages (Aramaki et al., 2013; Aramaki et al., 2021; Chen et al., 2017; Kojima et al., 2017), suggesting that germ cell specification is not clearly separated from pan-mesodermal development but rather regulated by common mesodermal TFs at the onset of gastrulation. Therefore, when taking these results together with the consistent loss/gain of function in our in vitro analysis, we think that our microscopic observation of mutant embryos was properly done.
In accordance with the Reviewer's suggestion, we performed statistical quantification of the PGC number in Yap-mutant embryos. This revealed significantly fewer PGCs in Yap-mutant embryos (Fig. 4D) but no significant change in Yap heterozygous–mutant embryos (Fig. S3G).

10.To determine differences between the lines, the E5 EpiSCs should be included in the qPCR analysis in Fig 1B. This will help to identify if there are any differentially expressed genes in the EpiSCs and after differentiation. The authors note there are no differences in expression of genes between the E3 and T9 lines when assessed via qPCR, however the flow cytometry data from PGCLC differentiation appears quite different with much higher levels of SSEA1 expression seen in the T9 line compared to E3. Can the authors include this in the qPCR gene panel and determine if these differences are present within the EpiSC population.

Response 11. We would like to point out that Q-PCR in Fig. 1B was done with the sorted PGCLC population (integrin ß-3- and SSEA1-double-positive cells), but not with the whole aggregates derived from EpiSCs/EpiLCs, leading to no difference in expression of PGC-related genes derived from the same PGCLC fraction. In this context, we have modified the text (Lines 116-119), the Fig. 1A and B and their legends to clearly show the Q-PCR analysis of FACS-sorted cells.
In addition, we would like to emphasize that the collection and subsequent analysis of E5-derived PGCLCs was not feasible, as the induction rate of PGCLCs from non-GC-E5 EpiSCs is quite low (Fig. 1A).

11.The references within the introduction relating to the involvement of WNT/ß-Catenin signalling in germ cell development are overstated. Line 49-54 states;'In mice, a co-operative regulatory mechanism between the BMP/SMAD and WNT/ß-CATENIN signalling pathways has been studied extensively and demonstrated that these two pathways are critical for the induction of germ cells and other neighbouring somatic lineages in nascent mesoderm derived from pluripotent epiblast' The evidence for BMP signalling involvement in germ cell development is much stronger than that for WNT/ß-CATENIN and this should be reflected in the language used, as the authors propose these have a similar impact. Further, the references for WNT signalling involvement (Liu 1999, Huelskin 2000 and Winnier 1996) should not be cited here as they do not include germ cell phenotypes. The authors should rework this section of the introduction to more accurately reflect the literature on WNT and BMP involvement in germ cell development.

Response 12. As we agree with the Reviewer's comment: "BMP signalling involvement in germ cell development is much stronger than that for WNT/ß-CATENIN", we separately described the significant function of WNT signaling and cited relevant references in the revised manuscript (Lines 56-67). On the other hand, we would like to note to the Reviewer that recent studies have clearly shown that WNT/ß-CATENIN signaling and its well-defined downstream factor T/Brachyury are required for germ cell fate induction (Aramaki et al., 2013; Ohinata et al., 2009). It has also been suggested in more recent publications that T/Brachyury directly regulates germ cell determinants such as Blimp1 and Prdm14 in a context-dependent manner by cooperating with residual pluripotency factors (Aramaki et al., 2021). Furthermore, recent studies in human have demonstrated that mesodermal factors have a critical function in directly regulating germ cell fate (Chen et al., 2017; Kojima et al., 2017). These results strongly suggest that germ cell specification is not clearly separated from pan-mesoderm development, but rather commonly induced in the downstream of combinatorial mesodermal signaling pathways at the onset of gastrulation. Thus, considering that there is a closely related mechanism regulating germ cell fate and pan-mesoderm differentiation, we have cited the relevant studies indicating the function of WNT signaling molecules in mesoderm development.

© 2021. Published by The Company of Biologists under the terms of the Creative Commons Attribution License (https://creativecommons.org/licenses/by/4.0/).

1.Table S1 – To facilitate re-use of the data, this should be provided as an excel spreadsheet rather than PDF download

Response 13. We sincerely thank the Reviewer for pointing this out. We have presented the data as a spreadsheet in the revised manuscript.

Minor Points
2.Although the paper conclusions centre around the role of YAP in germline competency, this is not mentioned within the introduction. Inclusion of some background information within the introduction would be beneficial to the reader for contextualisation of the experiments.

Response 14. We sincerely thank the Reviewer for pointing this out. We have incorporated the background of Hippo-Yap signaling into the introduction (Lines 82-90 of text).

3.There have been some previous reports of YAP involvement in the germline. It would be useful to have these in the discussion to support the arguments for YAP involvement in this context. For example, in Ye (2017, PMID: 28245464) Yap1 was overexpressed and downregulated in ovarian germline stem cells (OGSCs). Germ cell markers were upregulated in Yap1 overexpressing OGSC, and mice showed follicular regeneration and increased birth rate. While shRNA knockdown led to reduced proliferation in cells, decreased follicules in mouse ovaries, and reduced birth rates indicating Yap involvement in ovarian germ cell development.

Response 15. We sincerely thank the Reviewer for pointing this out. We have included the indicated publication in the discussion (Lines 318-320 of text).

4.What are the main differences between the 3 EpiSC lines? Are they all from the same mouse background – this should be included in the text.

Response 16. E3 and E5 EpiSC lines were established in our laboratory (Greber et al., 2010) and have the same background, whereas T9 EpiSCs were established by other researchers and have a different background. We have incorporated more detailed information into the revised manuscript (Lines 379-381 of text).

5.In the PGCLC differentiation experiments there is no indication of how many times these have been performed or if the results are from a single experiment.

Response 17. In accordance with the Reviewer's suggestion, we have indicated the number of replicates in each figure legend for PGCLC induction (Fig. 1A, C, and D, Fig. 4A, Fig. 5A). In addition, we have presented the induction rate of PGCLCs in each replicate in the supplementary figures (Fig. S1B, D, and F-J, Fig. S3D, Fig. S4D).

6.Fig 1B –It is difficult to determine if these cells have been sorted prior to qPCR analysis.

Response 18. We sincerely thank the Reviewer for pointing this out. We modified Fig. 1A and B to clearly denote that our Q-PCR analysis was done in the FACS-sorted cell fraction of PGCLCs (i.e., integrin β-3- and SSEA1-double-positive cells).

7.Fig 1D – there is no EpiLC control for these lines.

Response 19. Please see Fig. S1H and I (Lines 142-144 of text), which indicates the control experiment of PGCLC induction from EpiLCs. In this experiment, EpiLC-derived PGCLCs were detected by Blimp1-RFP reporter, which equivalently detects PGCLCs recognized by immunostaining with anti-integrin β-3 and anti-SSEA1 (Hayashi et al., 2011).

8.Fig 3A, Fig 4E- These figures would be much more readable if turned 90 degrees.

Response 20. In accordance with the Reviewer's suggestion, we have rotated the figure of our clustering data by 90 degrees.

© 2021. Published by The Company of Biologists under the terms of the Creative Commons Attribution License (https://creativecommons.org/licenses/by/4.0/).

9.Fig 3A – the PGCLCs from E3 and T9 are more similar to in vivo PGCs than ESC derived PGCLCs than would be expected. Can the authors comment on why they think this is the case.

Response 21. We would like to note to the Reviewer that the transcriptome of ESC-derived PGCLCs clustered together with in vivo PGCs in a distinct branch away from that of EpiSC-derived PGCLCs (Fig. 3B).

10.Figure 4H – figure has a spelling error, should be 'nucleus'

Response 22.
We sincerely thank the Reviewer for pointing this out. We have corrected the indicated description.

11.Fig EV1 – The diagram includes GFP being added to the PGC differentiation media – this may be a typo? Can the authors also explain why ROCK inhibitor was used as this is not standard practice for mouse PGCLC differentiation.

Response 23. We sincerely thank the Reviewer for pointing out the typo. We have corrected the relevant description in the figure. Regarding the ROCK inhibitor Y27632, we use this chemical to improve the survival rate of EpiSCs during PGCLC induction according to the previous study (Watanabe et al., 2007), which we have described in the revised manuscript (Lines 413-415 of text).

12.Line 118 – the specific numbers of PGCLCs is given. This should be presented as a percentage of total cells as this is variable between samples.

Response 24. As the experiment was done with two independent replicates, we have now deleted the description of the specific numbers and percentage in one experiment indicated from the original text. Instead, we present a table indicating the cell number and the percentage of induced PGCLCs (Fig. S1G).

13.Line 177 – the authors should expand on what the component of the pathway they are referring to is.

Response 25. According to the Reviewer's suggestion, we have modified the description and described the concrete component of the relevant signaling pathways (BMP4/SMAD1 and WNT3/ß-CATENIN) by citing the relevant publications (Lines 204–209 of text).

14.Line 224 – The authors should clarify that they have shown YAP contributes to germ cell specification through WNT signalling in this specific context, rather than in general.

Response 26. As we have described in Response 12, we consider that germ cell specification is not clearly separated from the general mesodermal development but rather regulated under the common mesodermal induction pathway at the onset of gastrulation (Please also see Response 12 and Response 9 to the Reviewer 2 described above for a more detailed rationale and the related literature). We thus do not think it logical discriminate YAP function in germ cell specification from its function in the general mesodermal differentiation. To show this notion more clearly, we have incorporated a summary diagram (Fig. 5C) and a corresponding passage into the revised manuscript (Lines 53-67, 283-286, 336-349 of text).

15.Line 231 – Previous studies are alluded to but references are missing.

Response 27. We sincerely thank the Reviewer for pointing this out. We have incorporated the proper reference into the revised manuscript (Line 294 of text).

16.Line 297 – details should be published in this paper if it is published first, otherwise link to preprint.

Response 28. As the detailed derivation method has been published in a very recent publication (Aramaki et al., 2021), we have cited this article in the revised manuscript (Line 386 of text).

© 2021. Published by The Company of Biologists under the terms of the Creative Commons Attribution License (https://creativecommons.org/licenses/by/4.0/).

17.Line 379 - Should read RNA Sequencing

Response 29.
We sincerely thank the Reviewer for pointing this out. We have properly corrected the description in the revised manuscript (Line 496 of text).

Aramaki, S., Hayashi, K., Kurimoto, K., Ohta, H., Yabuta, Y., Iwanari, H., Mochizuki, Y., Hamakubo, T., Kato, Y., Shirahige, K., et al. (2013). A mesodermal factor, T, specifies mouse germ cell fate by directly activating germline determinants. Dev Cell 27, 516-529.
Aramaki, S., Kagiwada, S., Wu, G. M., Obridge, D., Adachi, K., Kutejova, E., Lickert, H., Hubner, K. and Scholer, H. R. (2021). Residual pluripotency is required for inductive germ cell segregation. Embo Rep.
Chen, D., Liu, W., Lukianchikov, A., Hancock, G. V., Zimmerman, J., Lowe, M. G., Kim, R., Galic, Z., Irie, N., Surani, M. A., et al. (2017). Germline competency of human embryonic stem cells depends on eomesodermin. Biol Reprod 97, 850-861.
Greber, B., Wu, G., Bernemann, C., Joo, J. Y., Han, D. W., Ko, K., Tapia, N., Sabour, D., Sterneckert, J., Tesar, P., et al. (2010). Conserved and divergent roles of FGF signaling in mouse epiblast stem cells and human embryonic stem cells. Cell Stem Cell 6, 215-226.
Hayashi, K., Ohta, H., Kurimoto, K., Aramaki, S. and Saitou, M. (2011). Reconstitution of the mouse germ cell specification pathway in culture by pluripotent stem cells. Cell 146, 519-532.
Kojima, Y., Sasaki, K., Yokobayashi, S., Sakai, Y., Nakamura, T., Yabuta, Y., Nakaki, F., Nagaoka, S., Woltjen, K., Hotta, A., et al. (2017). Evolutionarily Distinctive Transcriptional and Signaling Programs Drive Human Germ Cell Lineage Specification from Pluripotent Stem Cells. Cell Stem Cell 21, 517-532 e515.
Kurimoto, K., Yabuta, Y., Ohinata, Y., Shigeta, M., Yamanaka, K. and Saitou, M. (2008). Complex genome-wide transcription dynamics orchestrated by Blimp1 for the specification of the germ cell lineage in mice. Genes Dev 22, 1617-1635.
Morin-Kensicki, E. M., Boone, B. N., Howell, M., Stonebraker, J. R., Teed, J., Alb, J. G., Magnuson, T. R., O'Neal, W. and Milgram, S. L. (2006). Defects in yolk sac vasculogenesis, chorioallantoic fusion, and embryonic axis elongation in mice with targeted disruption of Yap65. Mol Cell Biol 26, 77-87.
Ohinata, Y., Ohta, H., Shigeta, M., Yamanaka, K., Wakayama, T. and Saitou, M. (2009). A signaling principle for the specification of the germ cell lineage in mice. Cell 137, 571-584.
Ohinata, Y., Payer, B., O'Carroll, D., Ancelin, K., Ono, Y., Sano, M., Barton, S. C., Obukhanych, T., Nussenzweig, M., Tarakhovsky, A., et al. (2005). Blimp1 is a critical determinant of the germ cell lineage in mice. Nature 436, 207-213.
Reginensi, A., Scott, R. P., Gregorieff, A., Bagherie-Lachidan, M., Chung, C., Lim, D. S., Pawson, T., Wrana, J. and McNeill, H. (2013). Yap- and Cdc42-dependent nephrogenesis and morphogenesis during mouse kidney development. PLoS Genet 9, e1003380.
Sugimoto, M. and Abe, K. (2007). X chromosome reactivation initiates in nascent primordial germ cells in mice. PLoS Genet 3, e116.
Watanabe, K., Ueno, M., Kamiya, D., Nishiyama, A., Matsumura, M., Wataya, T., Takahashi, J. B., Nishikawa, S., Nishikawa, S., Muguruma, K., et al. (2007). A ROCK inhibitor permits survival of dissociated human embryonic stem cells. Nat Biotechnol 25, 681-686.

Second decision letter

MS ID#: DEVELOP/2021/199732

MS TITLE: YAP creates epiblast responsiveness to inductive signals for germ cell fate

AUTHORS: Saya Kagiwada, Shinya Aramaki, Guangming Wu, David Obridge, Borami Shin, Kenjiro Adachi, Eva Kutejova, Jeffrey L. Wrana, Karin Hübner, and Hans R. Schöler

© 2021. Published by The Company of Biologists under the terms of the Creative Commons Attribution License (https://creativecommons.org/licenses/by/4.0/).

Thank you for resubmitting your above revised manuscript. Your manuscript was re-reviewed by the original three reviewers, and I have now received all the referees reports. The referees' comments are appended below, or you can access them online: please go to BenchPress and click on the 'Manuscripts with Decisions' queue in the Author Area.

The overall evaluation is positive and the reviewers found that you addressed most of the comments they raised in their original revision. The three reviewers, however, have some remaining concerns. While it is not typical of Development to go for a second round of revision,these points are sufficiently important for the reviewers so that we ask you to make all efforts to address them, but also relatively minor, in terms of experimental effort, so that we can be confident that you can address them prior to publication in Development.

In particular, I am highlighting the two points that must be addressed:

1. Reviewer 1 request to compare the transcriptome of your EpiSCs to that of the formative stem cells by Kinoshita et al, Cell Stem Cell, 2020). We do share the Reviewer's view that this is an important point.

2. Both Reviewer 3 and Reviewer 2 raised issues regarding the number of replicates quantified in the nuclear/cytoplasmic YAP ratio analysis, which need to be addressed.

In addition, if you have any available Q-RT-PCR data on consensus PGC markers during the first 4-6 days of PGCL differentiation that you could add to the manuscript, that would greatly sustain the claim that YAP overexpression rescues PGCLC differentiation. However, I am aware that this comes late in the reviewing process and I would understand if you do not have such data, in which case, it would be important to introduce a sentence to indicate, for the readers, that additional molecular characterisation may be performed in the future.

Please attend to all of the reviewers' comments in your revised manuscript and detail them briefly in your point-by-point response. If you do not agree with any of their criticisms or the suggestions I have made, please do not hesitate to contact me.

Reviewer 1

Advance summary and potential significance to field

In this revised manuscript, the authors made appropriate revision based on the reviewers' questions. In general, the quality of the manuscript is significantly improved and almost reaches to the level for publication in Development.

Comments for the author

This reviewer would likes to ask the authors to answer the remained request to compare the transcriptome of their EpiSCs to that of the formative stem cells (Kinoshita et al, Cell Stem Cell, 2020). Since the previous report stated that the gremlin competency is unique character of the formative stem cells, this is very important point to be addressed the similarity/difference between the formative stem cells and EpiSCs.

Reviewer 2

Advance summary and potential significance to field

In this manuscript Kagiwada et al. examine whether different epiblast stem cell lines are competent for mesodermal and primordial germ cell like cell fate specification and characterize the molecular underpinnings of the observed differences. They find that differential Yap activity is responsible for the observed developmental competence, and therefore reveal a novel role for Yap during gastrulation and germ cell specification.

© 2021. Published by The Company of Biologists under the terms of the Creative Commons Attribution License (https://creativecommons.org/licenses/by/4.0/).

Comments for the author

The authors have addressed most concerns in a satisfactory manner. Thank you for clarifying the relationship between mesoderm and germ cell fate specification.
There are two points however, that require clarification.
Response 4. In accordance with the Reviewer's suggestion, we quantified the N/C (nuclear/cytoplasmic) YAP ratios in the examined GC- or non-GC-EpiSC lines in the revised manuscript. This analysis revealed a higher nuclear localization of YAP in competent EpiSCs compared with the incompetent lines (Fig. 3E). Furthermore, we have also seen phospho-YAP protein together with pan-YAP in western blot analysis.
We found that both forms of YAP protein are clearly activated in GC-EpiSC lines (E3 and T9) but noticeably affected in non-GC E5 EpiSCs, suggesting that there is more active Hippo signaling and its driven degradation of YAP in non-GC EpiSCs. We have incorporated these data and a relevant passage into the revised manuscript (Fig. 3G, Lines 220-235 of text).
It is unclear if the N/C Yap rations were quantified from 3 separate images of the same staining or from 3 independent stainings. It should be from 3 independent stainings, as a small difference in say permeabilization can result in the observed difference in cytoplasmic Yap intensity.
Response 6. We consider that it is unclear whether there is a discrepancy between the forming ability of Yap-mutant PGCLCs (in vitro) and PGCs (in vivo) based on our findings here, as it is difficult to evaluate the equivalence of the developmental stage of Yap-mutant PGCs and PGCLCs. Our data actually shows that poor induction of PGCLCs is still observed in Yap-mutant EpiSCs (Fig. 4A) as well as in vivo PGCs in Yap-mutant embryos (Fig. 4B-D). Furthermore, additional Q-PCR analysis also indicates that expression of Taz and early germ cell determinants such as Blimp1 is not affected in Yap-mutant EpiSCs at early time points (day 0 2, and 4) after cytokine stimulation (Fig. S3H), suggesting that Taz or other factors may compensate for the competence in Yap-mutant EpiSCs. We have included a corresponding passage in the revised manuscript (Lines 327-332 of text).
This section is quite confusing. Based on Fig. S3H, Blimp, Prdm14 and Ap-2y are all induced at a similar level in GC-EpiSCs (E3) and Yap KO GC-EpiSCs (Yap KO E3) on day 2 and 4 of cytokine induction. However, on day 6 of induction there are very few integrin-B3 and SSEA1 positive PGCLCs induced from Yap KO E3 cells (Figure 4A). The authors state that "as the SSEA1/Integrin ß-3 double-positive cells are the population recognized as being equivalent to Blimp1 reporter-positive PGCLCs (Hayashi et al., 424 2011).", it is difficult to envision that between day 4 and 6 of differentiation of Yap KO E3s all PGC marker expression disappears. Please include a full time course of differentiation with Blimp, Prdm14, Ap-2y and integrin-B/SSEA1 for E3 wt and E3 Yap KO cells.
Please discuss this in the results section, currently the data in Figure S3H is only mentioned in the discussion.

Reviewer 3

Advance summary and potential significance to field

This paper proposes a role for YAP in germ cell competence based on the germ cell competence of distinct EpiSC lines.

Comments for the author

The authors have addressed most concerns. However, there are a couple of points concerning the new data on the YAP overexpression rescue that could further strengthen the manuscript.
Figure 5A shows the results for b-integrin / SSEA1 expression following YAP overexpression in the non-germcell competent line E5 (performed twice).
Figure 5B shows that YAP overexpression increases expression of T and Eomes during the first 2 days of PGCLC differentiation (performed 3 times).
As the authors claim is that YAP overexpression rescues PGCLC differentiation, this claim should be supported by Q-RT-PCR analysis of the consensus PGC markers Blimp1, Prdm14 and Ap2g during the first 4-6 days of PGCLC differentiation. This should be done at least 3 times and the data analysed statistically.

© 2021. Published by The Company of Biologists under the terms of the Creative Commons Attribution License (https://creativecommons.org/licenses/by/4.0/).

In the authors response #9 they say they quantified the nuclear/cytoplasmic ratio of YAP using 3 independent images in each EpiSC line. Does this mean that 3 separate fields of view were assessed from the same culture of each line? It would be helpful if the authors could be explicit. If this is 3 fields of view from the same culture, the analysis should be repeated on separate, independent passages of the three EpiSC lines, to provide some confidence that the result is reproducible and not due to differences in the density of the E5 line compared to the E3 and T9 lines, as currently appears to be the case from Fig 2D.
These data (Fig 3 D, F) suggest that the nuclear/cyoplasmic ratio of YAP may influence nuclear size. It would therefore be informative if the authors could measure the nuclear/cytoplasmic ration of E5 cells before and after YAP overexpression.

Second revision

Author response to reviewers' comments

Reviewer 1 Advance summary and potential significance to field
In this revised manuscript, the authors made appropriate revision based on the reviewers' questions. In general, the quality of the manuscript is significantly improved and almost reaches to the level for publication in Development.

Reviewer 1 Comments for the author
This reviewer would likes to ask the authors to answer the remained request to compare the transcriptome of their EpiSCs to that of the formative stem cells (Kinoshita et al, Cell Stem Cell, 2020). Since the previous report stated that the gremlin competency is unique character of the formative stem cells, this is very important point to be addressed the similarity/difference between the formative stem cells and EpiSCs.
In the revised manuscript, we have included the transcriptomic analysis data and a relevant passage (Fig. S1E, lines 125–132 of text), indicating that our EpiSC models exhibit transcriptomic profiles more similar to EpiLCs than to the formative stem cells (FS cells) and EpiSCs cultured under the original Hayashi's condition.

Reviewer 2 Advance summary and potential significance to field
In this manuscript Kagiwada et al. examine whether different epiblast stem cell lines are competent for mesodermal and primordial germ cell like cell fate specification and characterize the molecular underpinnings of the observed differences. They find that differential Yap activity is responsible for the observed developmental competence, and therefore reveal a novel role for Yap during gastrulation and germ cell specification.

Reviewer 2 Comments for the author
The authors have addressed most concerns in a satisfactory manner. Thank you for clarifying the relationship between mesoderm and germ cell fate specification.
There are two points however, that require clarification.

Response 4. In accordance with the Reviewer's suggestion, we have quantified the N/C (nuclear/cytoplasmic) YAP ratios in the examined GC- or non-GC-EpiSC lines in the revised manuscript. This analysis revealed a higher nuclear localization of YAP in competent EpiSCs compared with the incompetent lines (Fig. 3E). Furthermore, we have also observed bands of phospho-YAP protein together with pan-YAP in western blot analysis. We found that both forms of YAP protein are clearly activated in GC-EpiSC lines (E3 and T9) but noticeably affected in non-GC E5 EpiSCs, suggesting that there is more active Hippo signaling and its driven degradation of YAP in non-GC EpiSCs. We have incorporated these data and a relevant passage into the revised manuscript (Fig. 3G, lines 220–235 of text).

It is unclear if the N/C Yap rations were quantified from 3 separate images of the same staining or from 3 independent stainings. It should be from 3 independent stainings, as a small difference in say permeabilization can result in the observed difference in cytoplasmic Yap intensity.

© 2021. Published by The Company of Biologists under the terms of the Creative Commons Attribution License (https://creativecommons.org/licenses/by/4.0/).

In the revised manuscript, we have described that the analysis was done in the images from 3 independent staining analyses (Lines 507–508, 838–839 of text).

Response 6. We consider that it is unclear whether there is a discrepancy between the forming ability of Yap-mutant PGCLCs (in vitro) and PGCs (in vivo) based on our findings here, as it is difficult to evaluate the equivalence of the developmental stage of Yap-mutant PGCs and PGCLCs. Our data shows that poor induction of PGCLCs is still observed in Yap-mutant EpiSCs (Fig. 4A) as well as in vivo PGCs in Yap-mutant embryos (Fig. 4B-D). Furthermore, additional Q-PCR analysis also indicates that expression of Taz and early germ cell determinants such as Blimp1 is not affected in Yap-mutant EpiSCs at early time points (days 0, 2, and 4) after cytokine stimulation (Fig. S3H), suggesting that Taz or other factors may compensate for the competence in Yap-mutant EpiSCs. We have included a corresponding passage in the revised manuscript (Lines 327–332 of text).

This section is quite confusing. Based on Fig. S3H, Blimp, Prdm14 and Ap-2y are all induced at a similar level in GC-EpiSCs (E3) and Yap KO GC-EpiSCs (Yap KO E3) on day 2 and 4 of cytokine induction. However, on day 6 of induction there are very few integrin-B3 and SSEA1 positive PGCLCs induced from Yap KO E3 cells (Figure 4A). The authors state that "as the SSEA1/Integrin ß-3 double-positive cells are the population recognized as being equivalent to Blimp1 reporter-positive PGCLCs (Hayashi et al., 424 2011).", it is difficult to envision that between day 4 and 6 of differentiation of Yap KO E3s all PGC marker expression disappears. Please include a full time course of differentiation with Blimp, Prdm14, Ap-2y and integrin-B/SSEA1 for E3 wt and E3 Yap KO cells.
Please discuss this in the results section, currently the data in Figure S3H is only mentioned in the discussion.

In accordance with the Reviewer's suggestion, we have included the data of day 6 of differentiation of Yap KO E3 (Fig. S3H). Consistent with FACS analysis, the qPCR analysis revealed that the expression of Blimp1 and Prdm14, two representative determinants, was affected in Yap KO E3 on day 6 compared with wild-type E3 or at other time points (days 2 and 4), whereas AP-2γ expression was not appreciably changed. We have incorporated a corresponding passage into the result section of the revised manuscript (Lines 246–249 of text).

Reviewer 3 Advance summary and potential significance to field This paper proposes a role for YAP in germ cell competence based on the germ cell competence of distinct EpiSC lines.

Reviewer 3 Comments for the author
The authors have addressed most concerns. However, there are a couple of points concerning the new data on the YAP overexpression rescue that could further strengthen the manuscript.

Figure 5A shows the results for b-integrin / SSEA1 expression following YAP overexpression in the non-germcell competent line E5 (performed twice).
Figure 5B shows that YAP overexpression increases expression of T and Eomes during the first 2 days of PGCLC differentiation (performed 3 times).
As the authors claim is that YAP overexpression rescues PGCLC differentiation, this claim should be supported by Q-RT-PCR analysis of the consensus PGC markers Blimp1, Prdm14 and Ap2g during the first 4-6 days of PGCLC differentiation. This should be done at least 3 times and the data analysed statistically.

We would like the reviewer to note that the previous study clearly indicates that integrinb3/ SSEA1 double-positive cells are equivalent to the cell fraction of functional PGCLCs with further differentiation competence, and the methodology is a reliable method for identifying specified PGCLCs in the relevant field (Hayashi et al., 2011; Murakami et al., 2016; Zhang et al., 2018). Thus, we think that the rescue of competence by exogenous YAP has been properly demonstrated by the presented data and thus we have not performed additional qPCR experiments to provide further supportive data. However, as we understand the significance of additional evaluation. such as molecular characterization and the related molecular mechanism, we have discussed this in the revised manuscript (Lines 345–348 of text).

© 2021. Published by The Company of Biologists under the terms of the Creative Commons Attribution License (https://creativecommons.org/licenses/by/4.0/).

In the authors response #9 they say they quantified the nuclear/cytoplasmic ratio of YAP using 3 independent images in each EpiSC line. Does this mean that 3 separate fields of view were assessed from the same culture of each line? It would be helpful if the authors could be explicit. If this is 3 fields of view from the same culture, the analysis should be repeated on separate, independent passages of the three EpiSC lines, to provide some confidence that the result is reproducible and not due to differences in the density of the E5 line compared to the E3 and T9 lines, as currently appears to be the case from Fig 2D.

In the revised manuscript, we have described that the analysis was done in the images from 3 independent staining analyses (Lines 507–508, 838–839 of text).

These data (Fig 3 D, F) suggest that the nuclear/cyoplasmic ratio of YAP may influence nuclear size. It would therefore be informative if the authors could measure the nuclear/cytoplasmic ration of E5 cells before and after YAP overexpression.
Although the effect on nuclear size by YAP overexpression in E5 is informative, we think that the suggested point regarding the nuclear size is not directly related to our main claim for competence restoration by exogenous YAP, which is sufficiently supported by the presented data. However, as we also think that more detailed mechanistic analyses, like the suggested point, would be informative for some readers in the future. We have thus mentioned the importance of additional evaluation such as molecular characterization and the related molecular mechanism in the discussion (Lines 345–348 of text).

References
Kinoshita, M., Barber, M., Mansfield, W.,Cui, Y., Spindlow, D., Stirparo, G. G., Dietmann, S., Nichols, J., Smith, A. (2021). Capture of Mouse and Human Stem Cells with Features of Formative Pluripotency. Cell Stem Cell 28, 453-471 e8.
Hayashi K, Ohta H, Kurimoto K, Aramaki S, Saitou M (2011) Reconstitution of the mouse germ cell specification pathway in culture by pluripotent stem cells. Cell 146: 519-32
Murakami K, Gunesdogan U, Zylicz JJ, Tang WWC, Sengupta R, Kobayashi T, Kim S, Butler R, Dietmann S, Surani MA (2016) NANOG alone induces germ cells in primed epiblast in vitro by activation of enhancers. Nature 529: 403-407
Zhang J, Zhang M, Acampora D, Vojtek M, Yuan D, Simeone A, Chambers I (2018) OTX2 restricts entry to the mouse germline. Nature 562: 595-599

Third decision letter

MS ID#: DEVELOP/2021/199732

MS TITLE: YAP creates epiblast responsiveness to inductive signals for germ cell fate

AUTHORS: Saya Kagiwada, Shinya Aramaki, Guangming Wu, Borami Shin, Eva Kutejova, David Obridge, Kenjiro Adachi, Jeffrey L. Wrana, Karin Hübner, and Hans R. Schöler
ARTICLE TYPE: Research Article

Thank you very much for submitting your revised manuscript, and for addressing the remaining concerns of the reviewers and the editor. I am happy to tell you that your manuscript has been accepted for publication in Development, pending our standard ethics checks.

© 2021. Published by The Company of Biologists under the terms of the Creative Commons Attribution License (https://creativecommons.org/licenses/by/4.0/).
